# Genome-wide identification and characterization of cation-proton antiporter (CPA) gene family in rice (*Oryza sativa* L.) and their expression profiles in response to phytohormones

**DOI:** 10.1371/journal.pone.0317008

**Published:** 2025-01-24

**Authors:** Md. Shohel Ul Islam, Nasrin Akter, Fatema Tuz Zohra, Shuraya Beente Rashid, Naimul Hasan, Shaikh Mizanur Rahman, Md. Abdur Rauf Sarkar

**Affiliations:** 1 Laboratory of Functional Genomics and Proteomics, Department of Genetic Engineering and Biotechnology, Faculty of Biological Science and Technology, Jashore University of Science and Technology, Jashore, Bangladesh; 2 Department of Genetic Engineering and Biotechnology, Faculty of Biological Sciences, University of Rajshahi, Rajshahi, Bangladesh; University of Delhi, INDIA

## Abstract

The cation-proton antiporter (CPA) superfamily plays pivotal roles in regulating cellular ion and pH homeostasis in plants. To date, the regulatory functions of CPA family members in rice (*Oryza sativa* L.) have not been elucidated. In this study, we use rice public data and information techniques, 29 *OsCPA* candidate genes were identified in the rice japonica variety (referred to as *OsCPA*) and phylogenetically categorized into K^+^ efflux antiporter (KEA), Na^+^/H^+^ exchanger (NHX), and cation/H^+^ exchanger (CHX) groups containing 4, 7, and 18 *OsCPA* genes. The OsCPA proteins were predominantly localized in the plasma membrane and unevenly scattered on 11 chromosomes. The structural analysis of OsCPA proteins revealed higher similarities within groups. Prediction of selection pressure, collinearity, and synteny analysis indicated that all duplicated *OsCPA* genes had undergone strong purifying selection throughout their evolution. The *cis*-acting regulatory elements (CAREs) analysis identified 56 CARE motifs responsive to light, tissue, hormones, and stresses. Additionally, 124 miRNA families were identified in the gene promoters, and *OsNHX7* was targeted by the highest number of miRNAs (43 miRNAs). Gene Ontology analysis demonstrated the numerous functions of *OsCPA* genes associated with biological processes (57.14%), cellular components (7.94%), and molecular functions (34.92%). A total of 12 transcription factor families (TFFs), including 40 TFs were identified in gene promoters, with the highest numbers of TFFs (5TFFs) linked to *OsCHX13*, and *OsCHX15*. Protein-protein interaction analysis suggested maximum functional similarities between rice and *Arabidopsis* CPA proteins. Based on expression analysis, *OsKEA1*, *OsKEA2*, *OsNHX3*, and *OsNHX7* were frequently expressed in rice tissues. Furthermore, *OsNHX3*, *OsNHX4*, *OsNHX6*, *OsNHX7*, *OsCHX8*, and *OsCHX17* in abscisic acid, *OsKEA1*, *OsNHX3*, and *OsCHX8* in gibberellic acid, *OsKEA1*, *OsKEA3*, *OsNHX1*, and *OsNHX3* in indole-3-acetic acid treatment were demonstrated as potential candidates in response to hormone. These findings highlight potential candidates for further characterization of *OsCPA* genes, which may aid in the development of rice varieties.

## 1.0 Introduction

The physiological processes of plants depend on intracellular ion and pH homeostasis within cellular organelles. The ion homeostasis process is regulated by various ion transporters, present in the organelle membranes, particularly in the plasma membrane. CPA is the most prevalent transmembrane transporter protein, regulating cytoplasmic ion concentration and pH homeostasis in plants by transporting cations and protons across the cell membrane [1]. The CPA protein family is classified into two major subfamilies: CPA1 and CPA2. In plants, the CPA1 subfamily includes Na^+^/H^+^ exchanger (NHX), which possess 10–12 membrane-spanning domains. The CPA2 subfamily comprises K^+^ efflux antiporter (KEA) and cation/H^+^ exchanger (CHX), having 8–14 membrane-spanning domains [2]. The CPA1 subfamily is associated with cellular growth, stress responses, protein processing, and vesicular trafficking while the CPA2 subfamily is involved in membrane trafficking and regulates enzyme activities [3].

The identification of the monovalent CPA proteins has widely been explored in various plant species, such as *Arabidopsis* (*Arabidopsis thaliana*) [4], pear (*Pyrus bretschneideri*) [5], and grape (*Vitis vinifera*) [6]. However, information on the transcriptomic expression profiles of these proteins in response to hormones remains lacking. Meanwhile, other gene families have been well-characterized regarding their hormonal response in various economically important plant species. For instance, most of the *OsIAA* genes in the rice genome are strongly induced in response to auxin and play crucial roles in auxin-mediated growth and development. Notable impacts were observed on *OsIAA9*, *OsIAA14*, *OsIAA19*, *OsIAA20*, *OsIAA24*, and *OsIAA31* as auxin elevated their transcript levels [7]. *OsWRKY* genes were induced by abscisic acid (ABA) and gibberellic acid (GA), demonstrating their involvement in the signaling pathway during seedling stages [8]. Additionally, the enhanced expression of *OsAGP1*, *OsAGP15*, and *OsELA3* by ABA (eNod-like AGP) implies that these genes may be stress-inducible and potentially contribute to the ABA signaling pathway [9]. The hormone-responsiveness spectrum of *OsUBC* revealed various induction kinetics of genes in response to indole-3-acetic acid (IAA), 6- benzylaminopurine (BA) [10].

Rice (*O*. *sativa* L.), the most cultivated cereal crop in the world is consumed by 3.5 billion people [11]. However, rice yield is declining due to numerous environmental anomalies, such as lower rainfall, cold weather, and higher salinity levels in soil. Additionally, rice production is hampered by diseases such as bacterial and fungal blight [12]. Uncovering the regulatory mechanisms of genes will benefit rice research and help meet global food demand. Rice also serves as an excellent model plant for genomics research and demonstrates the evolution of monocotyledon [13]. Technologies to identify hundreds of genes affecting essential agronomic aspects in rice have been improved rapidly. Genome-wide investigations using bioinformatics approaches are considered effective for revealing the functions of uncharacterized genes in various commercially important crops. However, to date, the CPA protein family in rice remains uncovered underscoring the importance of analyzing the functions of the OsCPA proteins.

In this study, we identified and characterized the CPA family in the rice genome through bioinformatics approaches. The OsCPA proteins were categorized into OsNHX, OsKEA, and OsCHX groups, and their protein properties were also investigated. Furthermore, we conducted phylogenetic analysis, gene structure, protein structure analysis, synonymous/non-synonymous analysis, collinearity analysis, synteny analysis, chromosomal distribution analysis, subcellular localization analysis, CARE analysis, miRNA-target site analysis, gene ontology analysis, transcription factor analysis, prediction of protein-protein interactions, the transcriptomic expression analysis in various developmental stages of tissues and also explored the significant expression profile of candidate genes in response to various phytohormones. Thus, our findings will provide an important clue for further research on the rice CPA protein family and in detail characterization of target genes to develop a new variety to meet the global demand of the increasing population.

## 2.0 Materials and methods

### 2.1 Database search and retrieval of CPA protein sequences in rice (*O*. *sativa*) genome

Initially, amino acid sequences of the *A*. *thaliana* were used as query sequences to retrieve CPA gene-encoding amino acid sequences in the *O*. *sativa* genome from Phytozome v13 (https://phytozome-next.jgi.doe.gov/) using BLASTp (Protein-basic local alignment search tool) [14], with default parameters. To confirm the presence of CPA proteins in the rice genome, the Pfam sodium/hydrogen ion (Na^+^/H^+^) exchanger domain "PF00999" was used as a query term. Subsequently, the retrieved protein sequences for CPA domains were analyzed using SMART (Simple Modular Architecture Research Tool, http://smart.embl-heidelberg.de/) [15], and the NCBI CDD (Conserved Domain Database) with default parameters (https://www.ncbi.nlm.nih.gov/Structure/cdd/wrpsb.cgi) [16]. Only the proteins containing the CPA-conserved domain (PF00999) were included in the candidate list. The OsCPA proteins were classified into OsKEA, OsNHX, and OsCHX groups according to the significant structural and functional similarity with the corresponding *Arabidopsis* protein sequences [17].

### 2.2 Determination of physio-chemical properties of OsCPA proteins

The ProtParam online tool (http://web.expasy.org/protparam/) was utilized to predict the basic physicochemical properties of OsCPA proteins such as amino acid residues (aa), molecular weight (MW), isoelectric point (pI), instability index, aliphatic index, and Grand Average of Hydropathicity (GRAVY) [18].

### 2.3 Phylogenetic analysis of CPA proteins of *Arabidopsis* (AtCPA) and rice (OsCPA)

The CPA protein sequences of *A*. *thaliana* and *O*. *sativa* were retrieved from Phytozome v13 (https://phytozome.jgi.doe.gov/pz/portal.html/), and aligned with the ClustalW program [19] (S1 Data). A phylogenetic tree was generated using MEGA11 software [20]. The Maximum Likelihood (ML) method was employed, including a 1000 bootstrap value to support values for each branch and Pearson correction. The constructed phylogenetic tree was then depicted using iTOL v6.7.4 (https://itol.embl.de/) [21].

### 2.4 Conserved domain analysis

Conserved domains of identified OsCPAs were predicted using the InterPro database (http://www.ebi.ac.uk/interpro/), and the results were illustrated using TBtools software-v1.116 [22].

### 2.5 Gene structure analysis

To analyze the gene structure of *OsCPAs*, coding sequences (CDS) and genomic DNA sequences in FASTA format, along with the "gf3" file of rice genome data were retrieved from the Phytozome v13 (https://phytozome.jgi.doe.gov/pz/portal.html/) (S2 and S3 Data). Gene Structure Display Server (GSDS v2.0) [23] available at http://gsds.cbi.pku.edu.cn/ was used to evaluate the gene structure of *OsCPAs*.

### 2.6 Conserved motif analysis

The conserved motifs of the OsCPA protein sequences were predicted utilizing the Multiple Expectation Maximization for Motif Elicitation program (MEME) (https://meme-suite.org/meme/tools/meme) (http://meme.nbcr.net/meme/) tools of MEME-suite (https://meme-suite.org/meme/) [24], with the number of motifs (20 motifs) with other default parameters (S4 Data). The motifs were then displayed using MEME and the motif scanning method (MSA), enabled through the MEME online interface.

### 2.7 Gene duplication analysis and synonymous (Ks) and non-synonymous (Ka) substitution ratios calculation

The Ka/Ks calculation tool (http://services.cbu.uib.no/tools/kaks) was used to determine the Ks and Ka substitution ratios of the duplicated *OsCPA* using CDC sequences. The rates of molecular evolution were analyzed using Ka/Ks ratios for each pair of paralogous genes. Additionally, the period of duplication and divergence (millions of years ago; MYA) was estimated using a synonymous mutation rate of substitutions per synonymous site per year as T = Ks/2λ×10^−6^ (λ = 6.5×10^−9^) [25].

### 2.8 Collinearity and synteny analysis of the *OsCPA* genes

To confirm gene duplication, collinearity and synteny analysis were performed using the Plant Genome Duplication Database online tool (http://chibba.agtec.uga.edu/duplication/index/locus) [26]. Additionally, TBtools version-v1.116 was performed to illustrate the *OsCPA* collinear pairs and their syntenic pairs with *Arabidopsis* (*A*. *thaliana*), maize (*Zea mays*), soybean (*Glycine max*), sorghum (*Sorghum bicolor*), and potato (*Solanum tuberosum*).

### 2.9 Analysis of the chromosomal location of *OsCPA* genes

The information on chromosomal length and location of *OsCPA* genes was collected from the Phytozome v13 database (https://phytozome.jgi.doe.gov/pz/portal.html/. The chromosomal localizations of *OsCPA* genes were mapped utilizing the MapGene2Chrom web v2 (MG2C) web server (http://mg2c.iask.in/mg2c_v2.0/) [27].

### 2.10 Prediction of the subcellular localization of OsCPA proteins

The subcellular localizations of the OsCPA proteins were predicted by the Wolf PSORT (https://wolfpsort.hgc.jp/) in various cellular organelles [28]. The OsCPA protein signals of each gene were illustrated by TBtools version-v1.116.

### 2.11 *Cis*-acting regulatory elements (CAREs) analysis of *OsCPA* gene promoters

To predict the CAREs, the 2000 bp 5’ untranslated regions (UTRs) of each *OsCPA* sequence were extracted from the Phytozome v13 database. Subsequently, CAREs of the *OsCPA* genes were analyzed using the plant CARE online tool (http://bioinformatics.psb.ugent.be/webtools/plantcare/html/) [29], and verified using PLACE databases (http://www.dna.afrc.go.jp/PLACE/) [30]. The predicted CAREs were categorized and the Chiplot online tool (https://www.chiplot.online/) was used to visualize the data.

### 2.12 Putative microRNA (miRNAs) target site analysis

The miRNAs were obtained from the plant microRNA encyclopedia (http://pmiren.com/) [31]. The CDS sequences of all *OsCPA* genes were examined for sequences complementary to miRNAs using the default parameters of psRNATarget (https://www.zhaolab.org/psRNATarget/analysis) [32] to identify miRNAs potentially targeting *OsCPA* genes.

### 2.13 Gene ontology (GO) analysis of *OsCPA* genes

GO analysis was conducted using the Plant Transcription Factor Database (PlantTFDB, https://planttfdb.cbi.pku.edu.cn//) to determine the relationship of *OsCPA* genes with various biological processes, cellular processes, and molecular functions [33].

### 2.14 Regulatory relationship between transcription factors (TFs) and *CPA* genes in rice

TFs in *OsCPA* gene promoters were identified using the PlantTFDB database. Furthermore, a regulatory network illustrating the interaction between *OsCPA* genes was constructed and TFs were predicted using Cytoscape 3.9.160 [34].

### 2.15 Protein-protein interaction (PPI) network prediction of OsCPA proteins

The PPI network of OsCPA proteins was analyzed using STRING version-11.0 (https://string-db.org/cgi/) database based on homologous protein from *Arabidopsis*. The PPI network analysis utilized four STRING tool parameters: (i) full STRING network, as network type, (ii) the meaning of network edge evidence, (iii) interaction score 0.4 (medium confidence parameter), and (iv) maximum number of interaction <10.

### 2.16 Expression pattern analysis of *OsCPA* genes in different tissues

RNA-seq data of the identified *CPA* genes in different tissues were obtained from the NCBI Sequence Read Archive (SRA) (https://www.ncbi.nlm.nih.gov/sra/). Subsequently, Trimmomatic package version 0.32 was used for quality control and trimming of transcriptomic data [35]. RNA sequencing was aligned to the rice reference genome from Phytozome v13 using the STAR package version 2.7.11b [36]. Samtools version 1.20 was performed for the conversion of sequence alignment map (SAM) files to binary alignment map (BAM) files, sorting, and arrangement [37]. RPKM (reads per kilobase million) values were used on a log2 transformed scale to represent the expression value. The Chiplot online tool (https://www.chiplot.online/) was used to visualize the retrieved data.

### 2.17 Expression analysis of *OsCPA* genes in response to phytohormones

The transcriptomic expression patterns of *OsCPA* genes of 15 days leaf tissues of three different rice varieties including Hanhui3 (HH3), Hanyou73 (HY73), and Huhan7A (HH7A) in response to ABA, GA, and IAA hormones, with control (HMCK), were analyzed using data retrieved from the NCBI SRA (https://www.ncbi.nlm.nih.gov/sra/). The Trimmomatic package version 0.32 was utilized for quality control and trimming of transcriptomic data. RNA sequencing was aligned to the rice reference genome from Phytozome v13 using the STAR package version 2.7.11b. Samtools version 1.20 was performed for the conversion of SAM files to BAM files, sorting, and arrangement. The RSEM package RSEM v1.1.17 was used to calculate fragments per kilobase million (FPKM) values of the transcriptomic data [38]. The Chiplot online tool (https://www.chiplot.online/) was used to visualize the heatmap.

## 3.0 Results and discussion

### 3.1 Identification and characterization of OsCPA proteins

A total of 29 CPA proteins were identified in the rice genome using AtCPA as references and classified into three groups: OsKEA (4 OsKEAs), OsNHX (7 OsNHXs), and OsCHX (18 OsCHXs). Since the CPA genes of *Arabidopsis* are more well-characterized than in other plant species and extensive bioinformatic tools and databases for *Arabidopsis*, such as The Arabidopsis Information Resource (TAIR: https://www.arabidopsis.org/), are available, we have used *A*. *thaliana* as query sequences. The number of CPA proteins in rice was higher than sorghum (*S*. *bicolor*) (28 CPAs), spreading earth moss (*Physcomitrella paten*) (22 CPAs), and green algae (*Chlamydomonas reinhardtii*) (10 CPAs) and lower as compared to grapevine (*V*. *vinifera*) (31 CPAs), maize (*Z*. *mays*) (33 CPAs), *Arabidopsis* (*A*. *thaliana*) (42 CPAs), black poplar (*Populus trichocarpa*) (44 CPAs), wheat (*Triticum aestivum*) (107 CPAs), and pear (*P*. *bretschneideri*) (53 CPAs) [39, 40].

Physio-chemical properties such as the size, MW (kDa), pI, instability index, aliphatic index, and GRAVY values of genes were also investigated (Table 1). The average length of OsKEA, OsNHX, and OsCHX proteins were 703.25 aa, 589.71 aa, and 771.17 aa residues, respectively. The protein containing comparatively larger amino acid lengths have complex structures with multiple domains and each domain might perform different functions while shorter proteins are simpler in structure with fewer domains [41]. Moreover, larger proteins have more regions for interacting with other proteins facilitating the formation of a larger network that enhances their functionalities in the cellular growth of plants [42]. The average molecular weights of these groups were 75.54, 65.18, and 82.38 kDa, respectively. The higher molecular weight of OsCPA proteins indicated their less solubility in an aqueous solution [43]. The pI values ranged from 5.03 to 8.57 (OsKEA2-OsKEA1), 5.6 to 8.67 (OsNHX5-OsNHX3), 4.91 to 9.27 (OsCHX2-OsCHX17), suggesting both acidic and basic properties of CPA proteins. The instability index of 17 OsCPA proteins (58.62%) was greater than 40 which were considered unstable proteins, whereas the remains were stable in various microenvironments. This stability is crucial for structural and functional studies of proteins. The GRAVY values reached 0.04 to 0.984 (OsCHX2-OsKEA1), indicating hydrophobic characteristics. In radish (*Raphanus sativus*), only 31.15% of RsCPA proteins were considered unstable while all RsCPA proteins were hydrophobic [2].

**Table 1:** List of 29 *OsCPA* genes and their basic physio-chemical characterization.

Gene Identifier	Gene name	Size (aa)	Molecular Weight (kDa)	pI	Instability Index	Aliphatic Index	Grand Average of Hydropathicity
LOC_Os03g03590	*OsKEA1*	241	26.76927	8.57	31.11	137.1	0.984
LOC_Os04g58620	*OsKEA2*	1154	12.38259	5.03	41.23	104.19	0.087
LOC_Os06g36590	*OsKEA3*	627	66.21287	5.69	32.08	124.7	0.681
LOC_Os12g42300	*OsKEA4*	791	85.34324	5.49	46.67	114.54	0.327
LOC_Os05g05590	*OsNHX1*	544	59.6444	7.17	34.66	121.65	0.68
LOC_Os06g21360	*OsNHX2*	528	58.21778	6.31	36.71	102.06	0.529
LOC_Os07g47100	*OsNHX3*	535	59.07039	8.67	35.58	107.89	0.556
LOC_Os09g11450	*OsNHX4*	549	60.39101	5.75	45.54	103.19	0.397
LOC_Os09g30446	*OsNHX5*	279	31.07192	5.6	41.33	105.95	0.415
LOC_Os11g42790	*OsNHX6*	545	59.9154	8.23	37.14	110.9	0.556
LOC_Os12g44360	*OsNHX7*	1148	12.791788	6.77	42.84	103.48	0.046
LOC_Os01g60140	*OsCHX1*	875	94.90454	6.34	40.4	109.95	0.419
LOC_Os01g73810	*OsCHX2*	258	27.58135	4.91	54.19	91.94	0.04
LOC_Os02g58660	*OsCHX3*	830	88.6908	6.43	37.25	101.57	0.315
LOC_Os03g61290	*OsCHX4*	780	82.322	8.84	39.28	111.58	0.437
LOC_Os05g02240	*OsCHX5*	844	89.6903	7.16	41.79	108.28	0.333
LOC_Os05g19500	*OsCHX6*	790	84.93739	7.02	38.99	112.33	0.41
LOC_Os05g31730	*OsCHX7*	453	46.15279	8.64	40.73	119.45	0.833
LOC_Os05g39600	*OsCHX8*	834	89.67656	8.78	48.09	108.36	0.374
LOC_Os05g40650	*OsCHX9*	874	93.26023	6.69	42.13	111.68	0.503
LOC_Os08g02450	*OsCHX10*	825	88.68029	6.33	48.23	106.21	0.398
LOC_Os08g43690	*OsCHX11*	817	87.82696	6.93	37.57	102.94	0.295
LOC_Os09g37300	*OsCHX12*	827	88.975	6.21	40.53	102.85	0.236
LOC_Os11g01820	*OsCHX13*	801	85.63571	6.75	40.72	108.93	0.447
LOC_Os11g03070	*OsCHX14*	822	87.23037	6.43	42.4	106.53	0.466
LOC_Os12g01820	*OsCHX15*	788	84.0959	7.6	39.84	108.24	0.441
LOC_Os12g02840	*OsCHX16*	839	89.04643	6.3	43	106.01	0.458
LOC_Os12g42200	*OsCHX17*	802	85.62558	9.27	42.37	109.88	0.431
LOC_Os12g44300	*OsCHX18*	822	88.44885	6.56	37.66	107.43	0.384

**Note:** GRAVY< 0, hydrophilic protein/GRAVY>0, hydrophobic protein [44], Instability Index (<40, the protein is stable/>40, the protein is unstable) [45].

### 3.2 Phylogenetic analysis of AtCPA and OsCPA proteins

**Fig 1:**
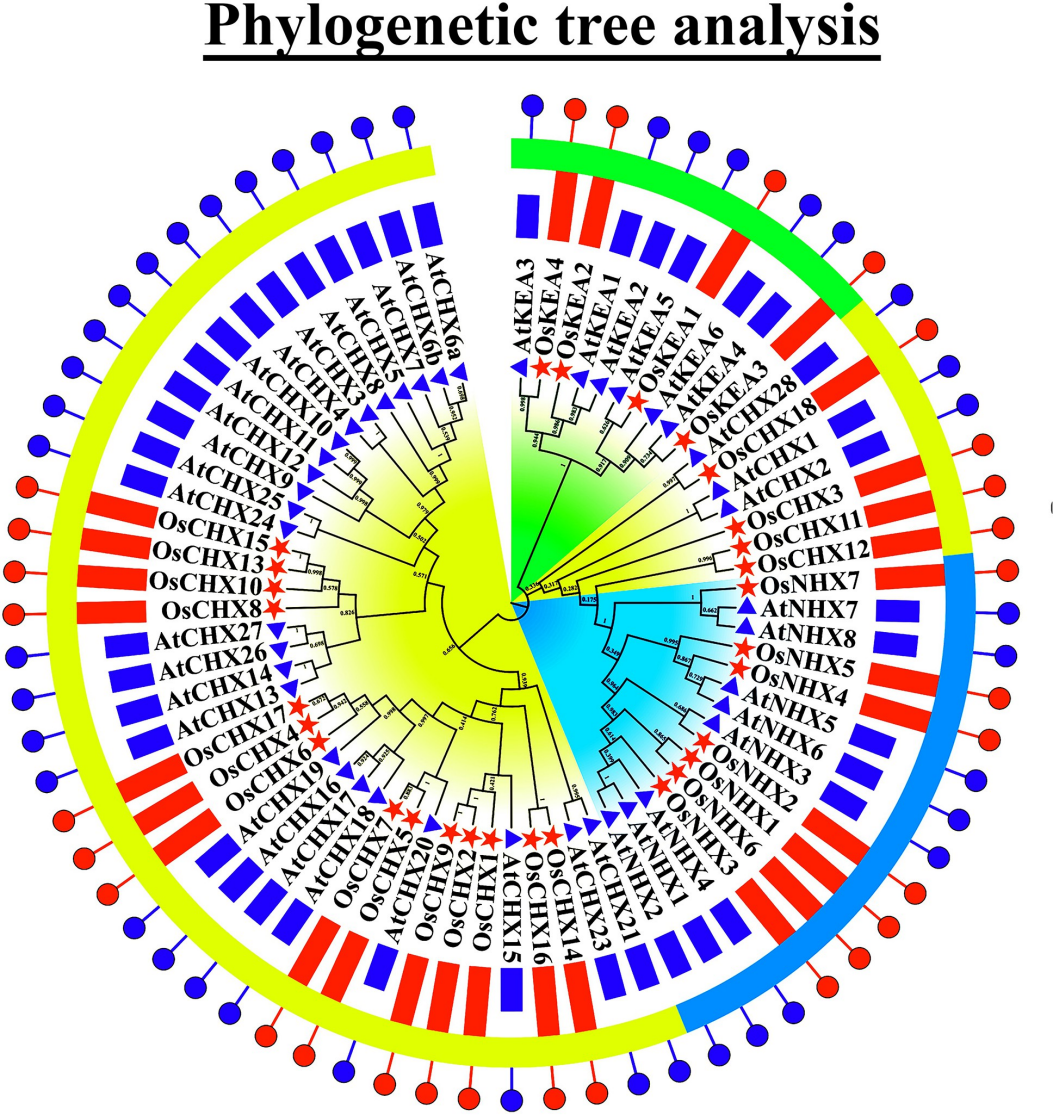
The phylogenetic relationship among 29 OsCPA and 42 AtCPA proteins. The CPA family was classified into 3 groups (KEA, NHX, and CHX). The KEA group is indicated by green color, the NHX group is indicated by turquoise color, and the CHX group is indicated by yellow color. The OsCPA and AtCPA genes are represented by a red star and blue triangle.

To investigate the evolutionary relationship among 29 OsCPA and 42 AtCPA proteins, a phylogenetic tree was constructed (Fig 1). The homologous CPA proteins from rice and *Arabidopsis* were highly clustered. For instance, OsCHX18, OsKEA1, OsKEA3, OsKEA4, and OsNHX2 were clustered with AtCHX28, AtKEA5, AtKEA4, AtKEA3, and AtNHX3, respectively. Some OsCPA proteins were grouped with multiple AtCPA proteins such as OsKEA2, OsNHX4, and OsNHX7, which clustered with AtKEA1, and AtKEA2; AtNHX5 and AtNHX6; AtNHX7 and AtNHX8, respectively. The clustered proteins are defined more through common ancestry, suggesting their similar functions due to duplication. Meanwhile, the CHX was the largest group, consisting of 46 CPAs from both rice and *Arabidopsis* while the KEA group was comprised of 10 CPAs and the NHX group contained 15 CPAs. The classification of OsCPA was consistent with the CPA protein family of various monocotyledons and dicotyledons plant species including grape, maize, and tomato [6, 46, 47]. This finding indicates that the classification of CPA proteins is conserved within species.

### 3.3 Conserved domain analysis of OsCPA proteins

**Fig 2:**
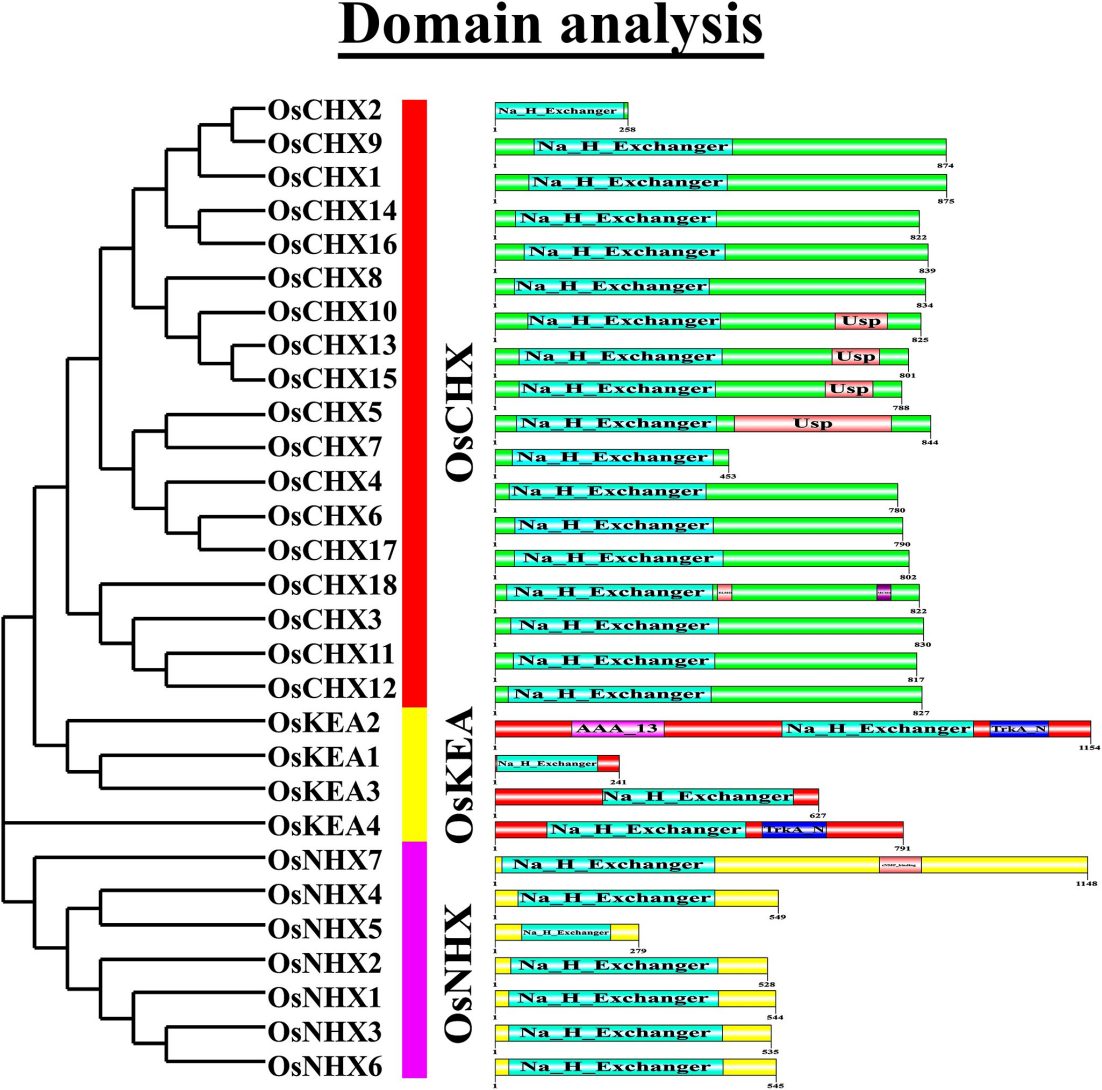
Distributions of conserved domains of OsCPA proteins. The relative positions of each domain are demonstrated in differently colored boxes, with corresponding domain names.

To gain insight into the protein structure and divergence, a conserved domain analysis of OsCPA proteins was conducted based on an evolutionary tree (Fig 2). In OsCHX groups, OsCHX5, OsCHX10, OsCHX13, and OsCHX15 proteins contained the ubiquitin-specific protease (USP; PF00582) as an additional domain with Na^+^/H^+^ exchanger domain (PF00999). The functional activity of USP domains is associated with phytohormone-regulated mechanisms and protects plants under stress [48]. For instance, the USP protein in *Arabidopsis*, controls the intracellular hydrogen peroxide concentration under hypoxic conditions and transmits the oxygen-deficient signal to the downstream defense mechanism [49]. Moreover, OsCHX18 contained an engulfment and cell motility domains (ELMO; PF11841) and a minichromosome maintenance-4 domain (MCM-4; PF00493). In the OsKEA group, OsKEA2 included a TrkA_N domain (PF02254), and AAA_13 (PF13166) as additional domains while OsKEA4 had only a TrkA_N domain (PF02254) along with Na^+^/H^+^ exchanger domain (PF00999). The TrkA-N is a NAD-binding domain that is essential for the proper functioning of transporters such as potassium channels [50]. Among the OsNHX group, the C-terminal of OsNHX7 possessed an additional cyclic nucleotide-monophosphate (cNMP; PF00027) binding domain. The cNMP domain plays a pivotal role in hormone signaling. For instance, treatment with GA on cereal aleurone layers causes an increase in cNMP levels, which then triggers the production of α-amylase, leading to the conversion of starch into sugar [51]. Additionally, auxin and kinetin, which are responsible for stomatal opening, also utilize this cNMP signaling pathway [52]. This study suggests potential functions of OsCPA proteins in plant development, hormone response, defense mechanism, and ion transportation.

### 3.4 Gene structure analysis of *OsCPA* genes

**Fig 3:**
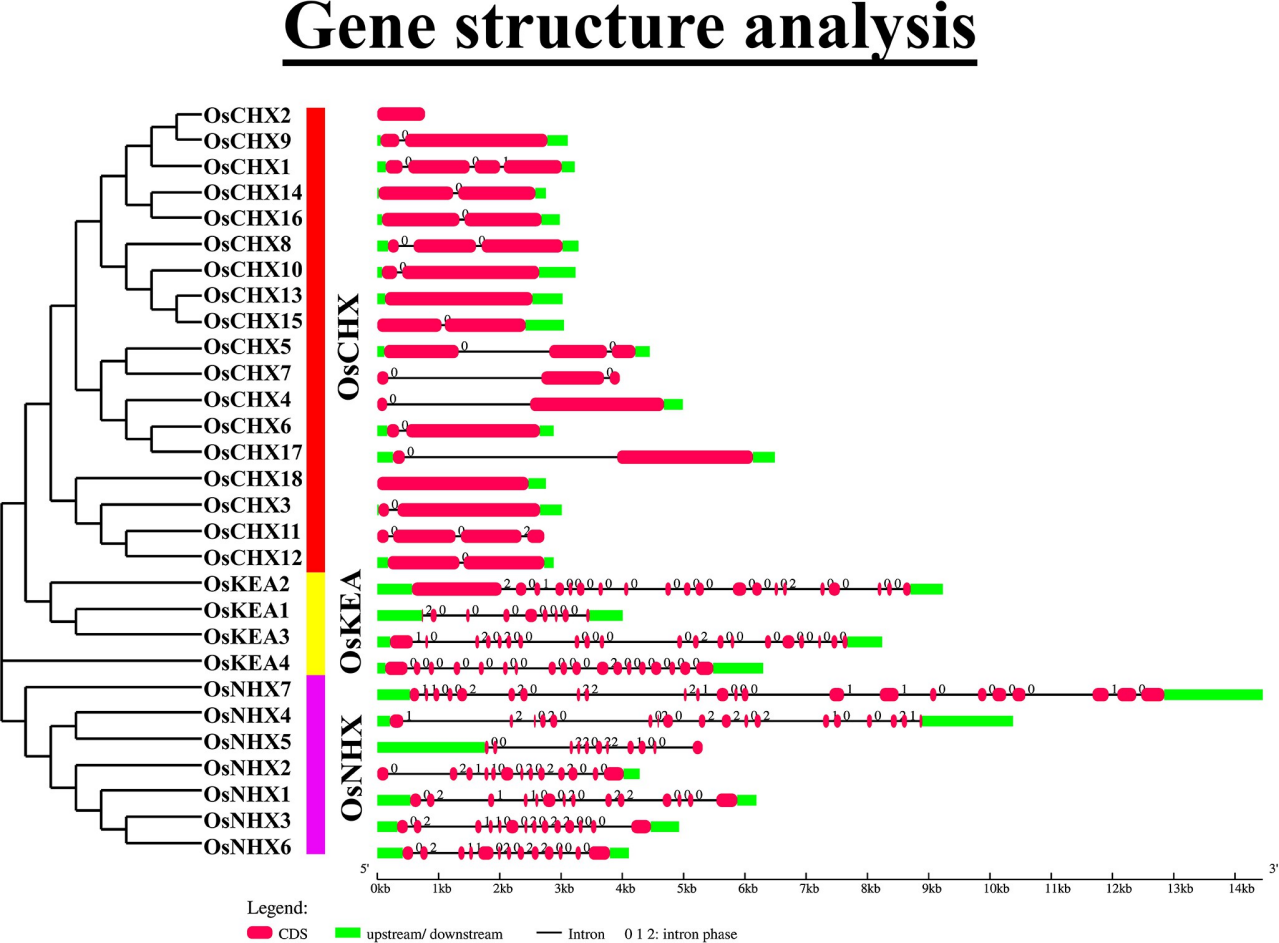
The gene structure of *OsCPAs*. For all *OsCPA* genes, black lines represent introns, pink-bold lines represent exons, and bright-green lines represent 5’ and 3’ UTRs. The exon/intron structure of each *OsCPA* gene is displayed proportionally according to the scale at the bottom.

To understand the structural information, the exon-intron organization of *OsCPAs* was analyzed based on evolutionary relationship (Fig 3 and S5 Data). As expected, *OsCPAs* within the same group exhibited similar gene structures, revealing a close evolutionary relationship among them. Significant differences in sequence length and the exon-intron number were also observed. *OsNHX7* shared the largest gene fragment (14 kb long) and the intron number varied from 0–22 in *OsCPAs*. *OsCHX2*, *OsCHX13*, and *OsCHX18* had no intron, suggesting their rapid responses to stress [53]. The average number of exons in *OsKEA*, *OsNHX*, and *OsCHX* were 16.75, 15.14, and 2.89, respectively. Interestingly, the *CHX2* gene contains only one exon without possessing introns and UTRs. The *CHX2* gene may be processed pseudogenes, as it lacks a 5ʹ promoter sequence. This gene may have lost intron over the evolutionary process and arisen through retroposition [54]. This gene lacks the complex regulatory mechanisms that simplify it in structure than other *CHX*. In wheat, exons numbers varied among these groups 12–21, 7–25, and 1–4 [46]; in radish, 17–21, 10–19, and 1–5 [2]; and in tomato, 7–20, 1–23, and 2–7 [47], respectively. The *OsCHX* genes had shorter gene segments with fewer exons and introns than the *OsKEA* and *OsNHX* genes. Additionally, different splicing methods might be responsible for variations in exon-intron combinations of *OsCPA* genes in evolution, leading to the production of proteins with particular functions to meet biochemical requirements. A similar phenomenon was also observed in pear, and *Arabidopsis* [5, 55]. This consistency in gene structure among various plant species suggests that *CPAs* are relatively conserved over evolution.

### 3.5 Conserved motif analysis of OsCPA proteins

**Fig 4:**
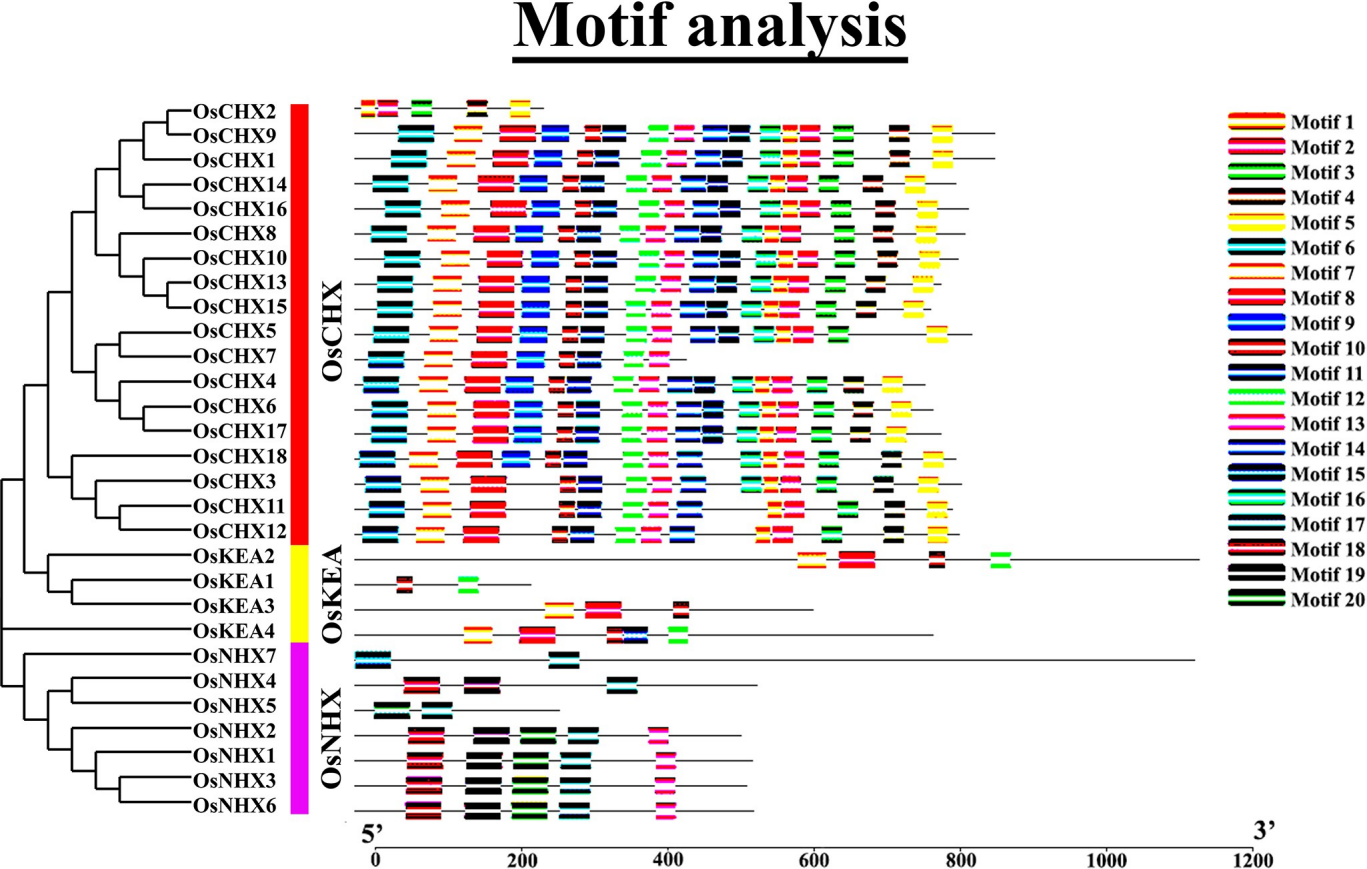
The distribution of conserved motifs in OsCPA proteins. The conserved motifs were identified in OsCPA proteins with a maximum number of 20. Each colored box aligned on the right side of the figure represents a specific motif.

A total of 20 conserved motifs were identified in OsCPA proteins, ranging from 2–16 in number (Fig 4). Motifs play a crucial role in gene regulation providing a binding site for transcription factors that control gene expression [56]. Most OsCPA proteins in the same group had conserved organization of motifs, suggesting their similarity in functions. The motif numbers varied across groups, such as OsCHXs were found to be the most abundant group, followed by OsNHXs and OsKEAs. In maize, ZmNHX proteins shared a maximum number of conserved motifs (5–8), while the ZmCHX proteins contained 6–7 conserved motifs [46]. Moreover, in potato, StCHX groups were more abundant than StKEA and StNHX proteins [57]. This suggests that the functions of different groups may have evolved. Notably, only motif 4 was identified in all groups indicating its conserved natures among all OsCPA proteins. Some motifs were unique to only one group. For instance, Motif 3, Motif 8, Motif 9, Motif 10, Motif 11, Motif 15, and Motif 16 were specific to the OsCHX group. Motif 17, Motif 18, Motif 19, and Motif 20 were distributed in only OsNHX group. Similar motif organizations were noticed in soybeans (*G*. *max* L) with some motifs being specific to certain groups [58]. In conclusion, OsCPA proteins shared similar motif organizations within species, and the motif numbers varied among groups that exhibited different functions.

### 3.6 Ka/Ks analysis of OsCPA gene family

**Fig 5:**
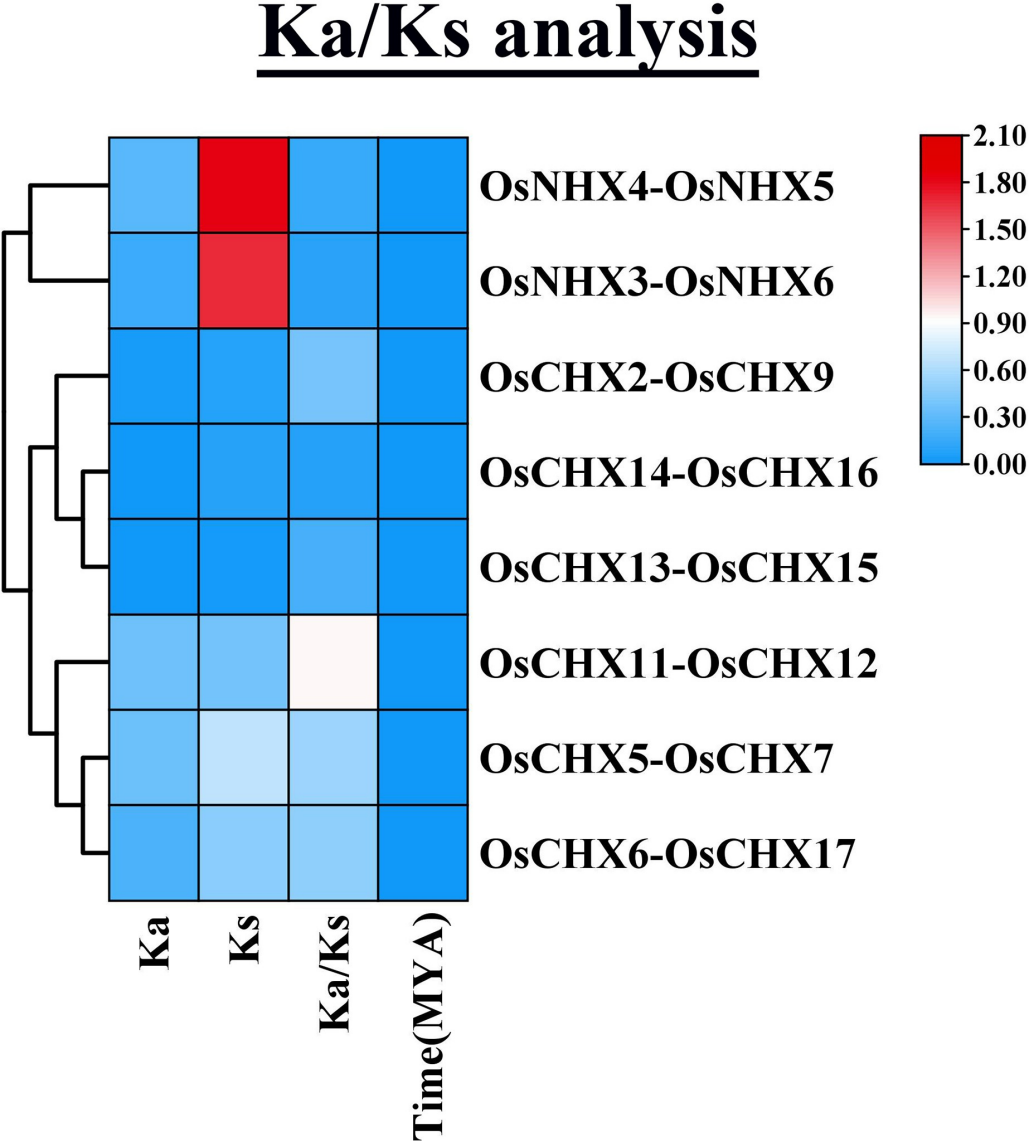
The estimation of gene duplication for different paralogous gene pairs among *OsCPA* genes based on Ka and Ks values. The number of non-synonymous substitutions per non-synonymous site is represented by Ka, while the number of synonymous substitutions per synonymous site is represented by Ks values. The ratio of Ka to Ks changes is represented by Ka/Ks. The color gradient on the right side represents the values ranging from blue to red (0 to 2.10).

To identify the existence of selective pressure including negative/purifying, neutral, and positive selections as well as the evolutionary relationships of *OsCPA* genes, Ka/Ks ratios for 8 *OsCPA* homologous pairs were calculated (Fig 5 and S6 Data). Selection pressure, influenced by external and internal factors, leads to reorganized genetic compositions in genes, resulting in the development of new traits within species [59]. The Ka/Ks ratios for *OsCPA* genes varied from 0.08 (*OsCHX14*-*OsCHX16*) to 0.94 (*OsCHX11*-*OsCHX12*) indicating that their evolution had undergone a strong purifying selective pressure. *CPA* genes in pear, radish, and sorghum also evolved through purifying selection [2, 5, 60]. Additionally, *OsNHXs* had lower average Ka/Ks ratios compared to *OsCHXs*, suggesting stronger purifying selection pressure on *OsNHX* genes. In soybean and radish, *KEA* genes experienced stronger purifying selection than *NHX* and *CHX* genes. However, the *GmNHXs* and *RsNHXs* underwent stronger purifying selection than *GmCHX* and *RsCHX*, respectively [2, 58]. The divergence period of duplicated *OsCPA* genes ranged from 7.32E-17 MYA (*OsCHX13*-*OsCHX15*) to 6.02E-15 MYA (*OsNHX4*-*OsNHX5*), with an average of 2.15E-15 MYA, demonstrating the more recent divergence events of *CPA* genes in rice compared to soybean (average 40.75 MYA) [58]. These results indicate a notable presence of strong purifying selection and retention of functions throughout the evolution of *OsCPA* genes.

### 3.7 Collinearity and synteny analysis of the *OsCPAs*

**Fig 6:**
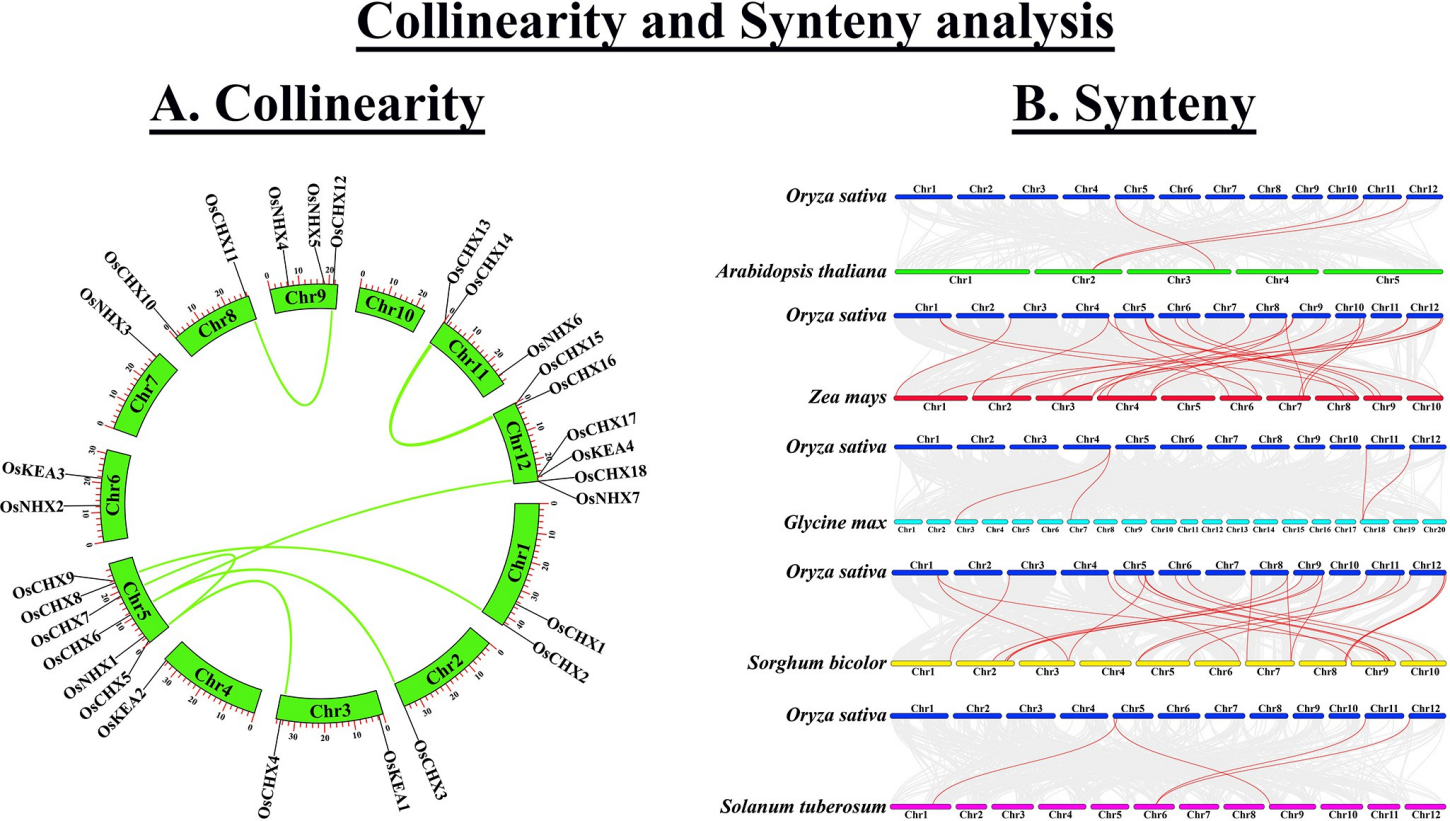
Collinearity and synteny analysis. **A.** The collinearity analysis of the *OsCPA* genes. *OsCPA* collinear blocks within the rice genome were represented by bright-green colored lines. The bright-green colored rectangles represent chromosomes 1–12. **B.** The synteny analysis of *CPA* genes between rice, *Arabidopsis*, maize, soybean, sorghum, and potato. The syntenic gene pairs of *OsCPA* are indicated by red colored lines. The chromosomes of different species are also represented by different colors.

Collinearity analysis was performed to reveal the possible regulatory functional activities of the *OsCPA* genes (Fig 6A). The collinearity analysis, a specific type of synteny analysis demands a highly conserved gene order [61]. This study identified 8 collinear pairs in the OsCPA family with the maximum number of collinear genes determined in Chr5 (6 collinear genes). However, Chr12 and Chr11 had three and two collinear genes, respectively, while Chr1, Chr2, Chr3, chr8, and Chr9 each contained only one collinear *OsCPA* gene. The predicted collinear pairs may be responsible for lineage-specific expansion throughout their evolutionary period [62]. Furthermore, to determine the evolution mechanisms and replication of the *OsCPA* genes, synteny analysis was conducted using synteny blocks (Fig 6B). Synteny analysis is fundamental for evolutionary investigations at the genome level and facilitates the gene annotation of newly sequenced genomes [63]. Based on the syntenic map, 3, 25, 4, 23, and 4 synteny gene pairs were observed in the *O*. *sativa-A*. *thaliana*, *O*. *sativa-Z*. *mays*, *O*. *sativa-G*. *max*, *O*. *sativa-S*. *bicolor*, and *O*. *sativa-S*. *tuberosum* CPA family, respectively. Synteny analysis was also performed for the CPA gene family in other plant species. For instance, 19, and 57 synteny pairs were found in grapevine-*Arabidopsis*, and radish-*Arabidopsis* [2, 6]. These analyses suggest that these paired genes might have a remarkable relationship in the context of duplication, evolution, function, and expression.

### 3.8 Analysis of the chromosomal location of *OsCPA* genes

**Fig 7:**
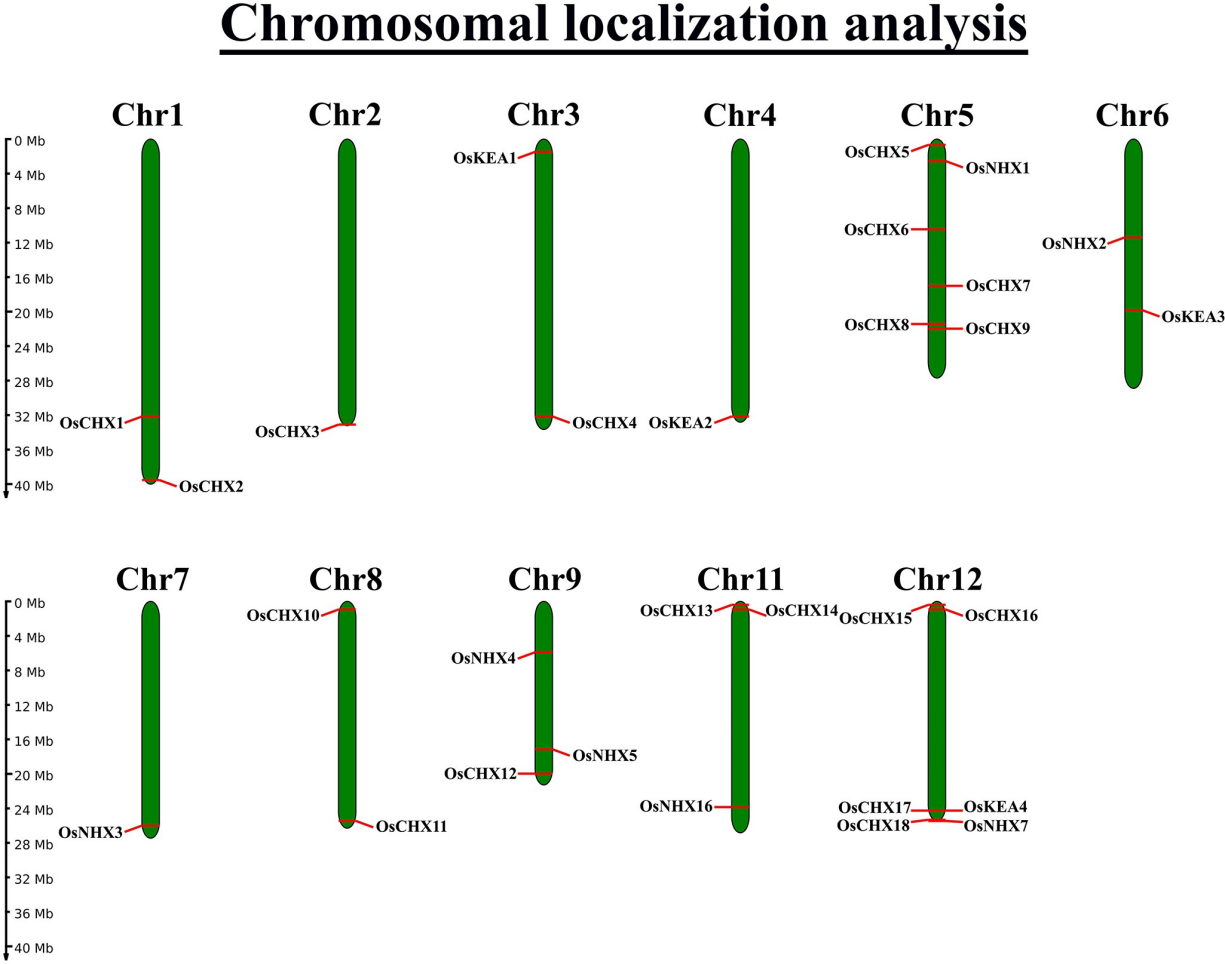
The chromosomal localizations of *OsCPA* genes. The chromosome numbers are aligned on the top of each chromosome bar. The chromosome-scale on the left-side represents the chromosome length.

To reveal the genomic positions of the predicted *OsCPA* genes, the chromosomal locations were investigated, demonstrating that 29 *OsCPA* genes were unevenly scattered on 11 of the 12 chromosomes (excluding Chr10) within a genomic length of 40 million bases (Mb) (Fig 7). The maximum number of genes (6 *OsCPA*) were distributed on Chr5 and Chr12 while only one gene was identified on Chr2, Chr4, and Chr7. Notably, longer chromosomes did not necessarily contain a higher number of *OsCPA* genes, suggesting no correlation between gene number and chromosomal length. The distribution of grape *CPA* genes supports our study, as *VvCPA* was also unevenly scattered on 14 of 19 chromosomes [6]. In soybean, potato, and radish, *CPA* genes were located on 20, 12, and 9 chromosomes, respectively [2, 57, 58]. Interestingly, maximum genes were located on the end position of the corresponding chromosomes suggesting potential contributions to genetic variations in traits [64].

### 3.9 Prediction of subcellular localization of OsCPA family members

**Fig 8:**
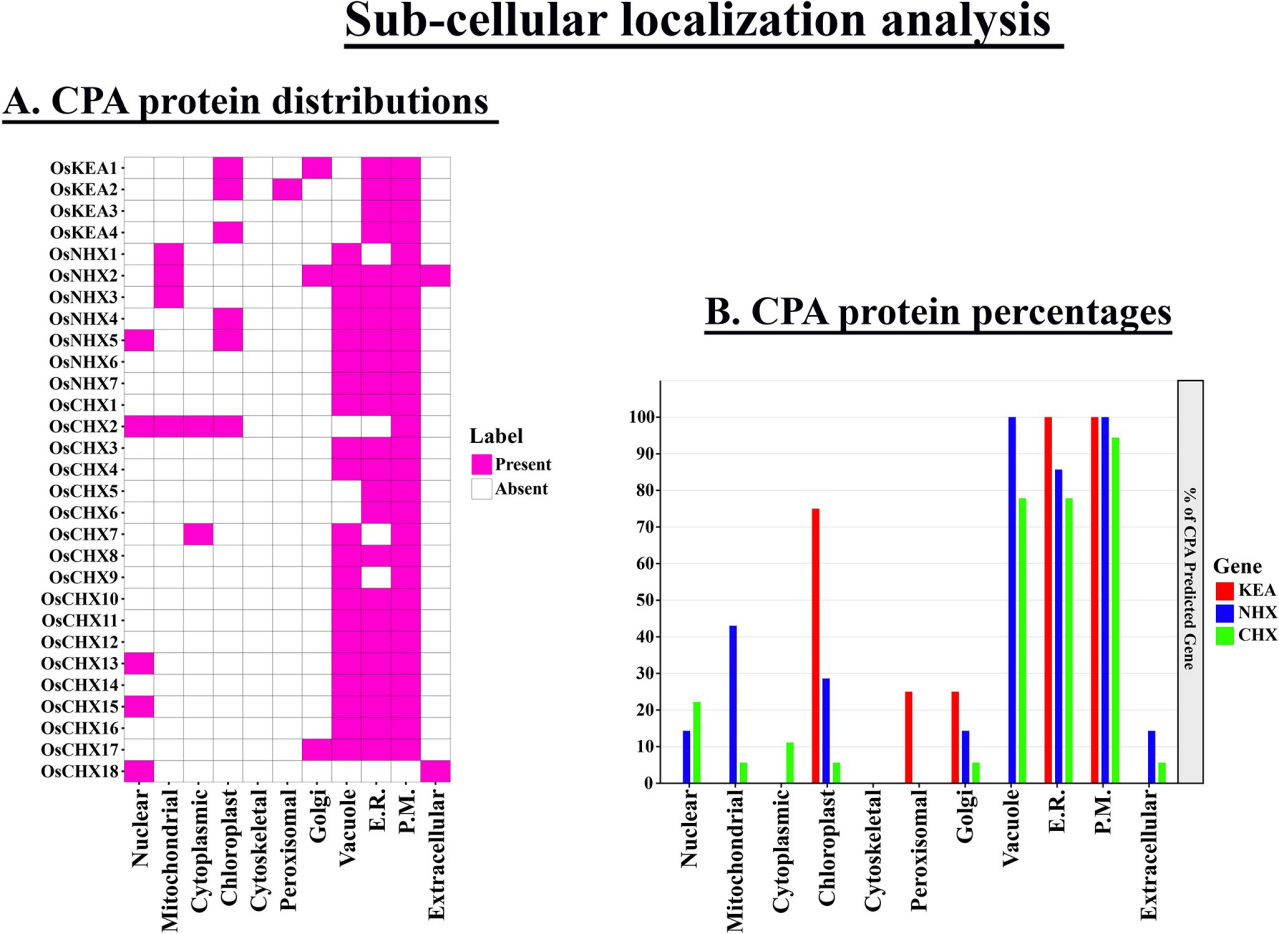
Sub-cellular localization analysis of OsCPA proteins. **A.** A heatmap represents the protein signals of OsCPA across various cellular organelles. The names of CPA proteins are shown on the left side of the heatmap, and the names of the corresponding cellular organelles are labeled at the bottom. The color intensity shown on the right side of the heatmap represents the presence of protein signals corresponding to the genes. **B.** The percentage of OsCPA protein signals across various cellular organelles is represented by a bar-plot. The percentages of protein signals appearing in different cellular organelles are shown on the left side of the bar-plot.

The prediction of subcellular localization provides insight into the cellular distribution of proteins and facilitates the investigation of their functions. OsCPA proteins were identified in various cellular organelles including the nucleus, mitochondria, cytoplasm, chloroplast, cytoskeletal, peroxisome, Golgi apparatus, vacuole, endoplasmic reticulum (E.R), plasma membrane (P.M), and extracellular region (Fig 8A and 8B). Most OsCPA protein signals were detected on the plasma membrane (96.55%)), followed by the endoplasmic reticulum (82.76%)) and vacuole (72.41%). The lowest prediction sites were observed in peroxisome accounted for 3.45% of OsCPA protein. The CPA proteins of potato, maize, and soybean were predominantly located on the plasma membrane, suggesting their possible functions in cell membrane connection, enhancing trans-membrane movements and plant response to various environmental stresses [46, 57, 58]. Additionally, the lowest prediction sites for StCPA were observed in the cytoplasm which supports our findings [57]. The vacuolar AtNHX1 enhances plant tolerance to salt by allowing Na^+^ transportation into the cytoplasm for sodium differentiation [65]. OsCPA proteins were assumed to be involved in the glycosylation mechanism, as well as in storing and transporting plant nutrients and metabolites, as they were also predominantly predicted on the endoplasmic reticulum. In *Arabidopsis* and maize, AtKEA1-3 and ZmKEA2-4 are localized in chloroplast, where they perceive stress signals and enhance the photosynthesis process [46, 66]. Chloroplast-localized OsCPA proteins contribute to maintaining structural integrity of chloroplast, and pH stability [67]. The variations in the localization of OsCPA proteins across different subcellular organelles suggest functional differences among these proteins.

### 3.10 *Cis*-acting regulatory elements (CAREs) analysis of *OsCPA* gene promoters

**Fig 9:**
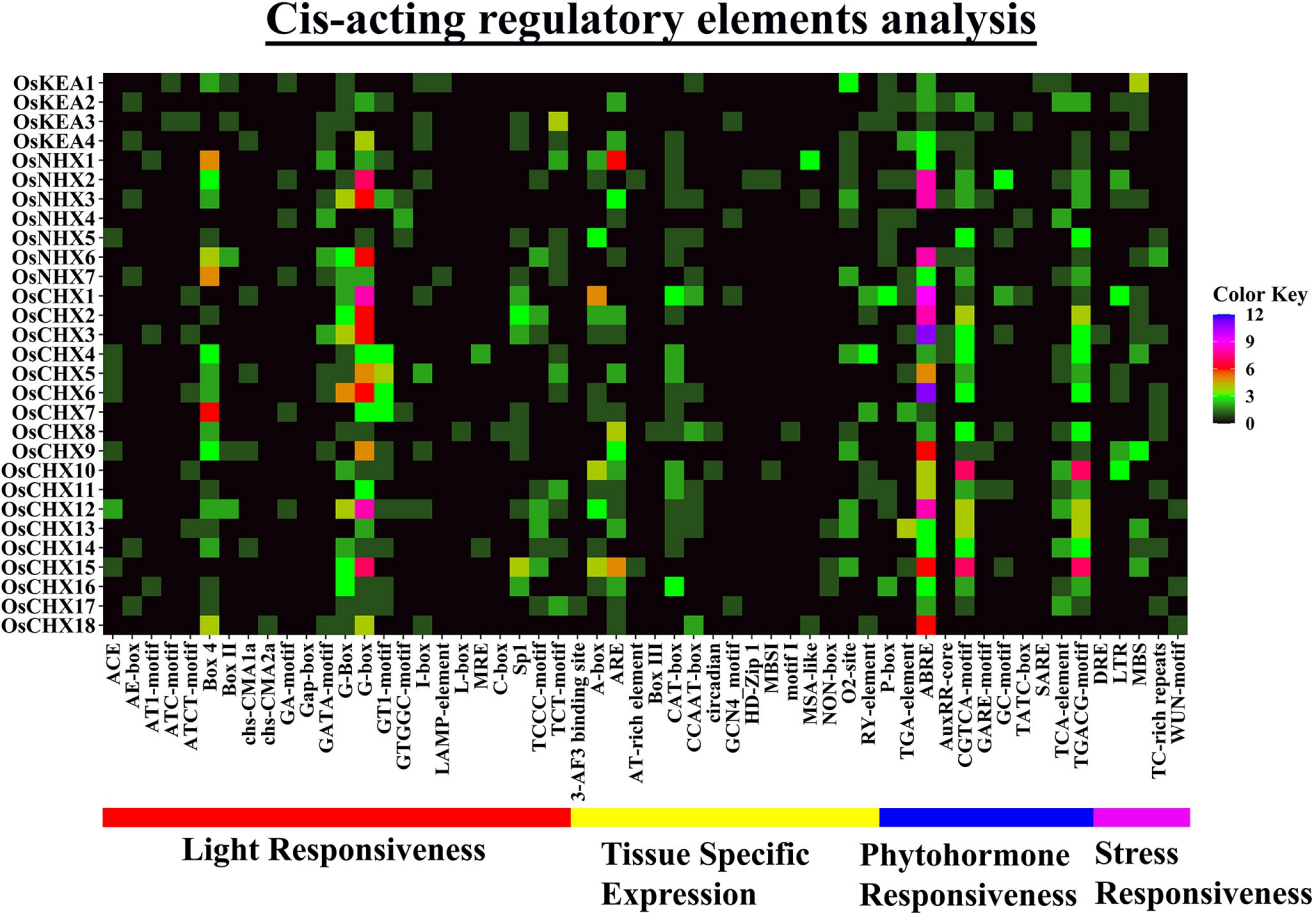
The distribution of putative CAREs on the 2.0 kb promoter region of *OsCPA* genes is represented by a heatmap. The names of each *OsCPA* gene are shown on the left side of the heatmap. The number of putative CAREs for each *OsCPA* gene is represented by four different color gradients (black = 0, green = 1–3, red = 4–6, pink = 7–9, and blue = 10–12). CAREs of the corresponding genes, associated with light responsiveness, tissue-specific expression, phytohormone responsiveness, and stress responsiveness are shown at the bottom of the heatmap and denoted by bold lines in red, yellow, blue, and pink, respectively.

CAREs are short DNA motifs (5–20 bp), identified in gene promoters that regulate gene expression in response to environmental alteration at the transcriptional level. A total of 56 CARE motifs were predicted in *OsCPA* gene promoters and classified into light responsiveness (24 motifs), tissue-specific expression (16 motifs), phytohormone responsiveness (11 motifs), and stress responsiveness (5 motifs) (Fig 9; S7 and S8 Data).

The light-responsive CAREs were abundantly present in *OsCPA* gene promoters, as previously observed in the CPA family of other economically important plant species such as maize and moso bamboo (*Phyllostachys edulis*) [46, 68]. G-box, Box-4, and G-Box were commonly detected in the *OsCPA* genes with G-box being highly prevalent in the upstream conserved region of *OsCHX*s. Light-responsive elements were abundant in *OsCHX12* (26), *OsCHX6* (20), *OsNHX6* (20), and *OsCHX5* (19), demonstrating their involvement in photosynthetic mechanisms, and gene regulation through light signals [69]. The G-box motif, activated by calcium-dependent phosphorylation and dephosphorylation processes, acts as a molecular switch in response to light and environmental stresses including injuries, UV light, and red light [70].

In an aspect of tissue-specific motifs, adenylate-uridylate-rich element (ARE) were the most commonly detected motif in *OsCPA* gene promoters. ARE motifs were present exclusively in *OsCHX* (except *OsCHX1*, *OsCHX4*, *OsCHX6*, and *OsCHX14*) than in *OsNHX*, and *OsKEA*. AREs regulate either cell growth or the impedance of a species to environmental factors. Tissue-specific motifs were abundant in *OsCHX1* (13), *OsCHX15* (13); *OsNHX1* (13), *OsCHX8* (11), and *OsCHX10* (11) suggesting their potential involvement in various biological functions including physiological growth and development of rice plant [71].

In terms of phytohormone-responsive elements, CARE motifs were particularly abundant in the *OsCHX12* (27) gene promoter followed by *OsCHX15* (22), *OsCHX10* (20), *OsCHX3* (20), *OsCHX1* (18), *OsNHX2* (18), and *OsCHX6* (17). ABA-responsive element (ABRE) was found more frequently in *OsCPA* genes except for *OsNHX4*, and *OsNHX5*, with abundance in *OsCHX3*, and *OsCHX6* (11 ABREs), which respond to dryness and salt signals [72]. Additionally, CGTCA-motif and TGACG-motif were also abundant in the *OsCPA* gene promoter, involved in Methyl jasmonate (MeJA) responsiveness [73].

The stress-responsive elements LTR and MBS were frequently identified in the *OsCPA* gene promoter and were highly present in *OsCHX1*, *OsCHX10*; and *OsKEA1*, respectively. The LTR motif is potentially associated with low temperature and stress response regulations while the MBS motif is essential for drought stress response [46, 74]. Nevertheless, 1–3 stress-responsive motifs were present in most of the *OsCPA* genes, *OsNHX1*, *OsNHX4*, and *OsNHX7* had no stress-responsive elements. The presence of various CAREs suggests differential functions of *OsCPA* genes in different tissues, light, hormonal, and stress conditions.

### 3.11 Putative microRNA (miRNAs) target site analysis

**Fig 10:**
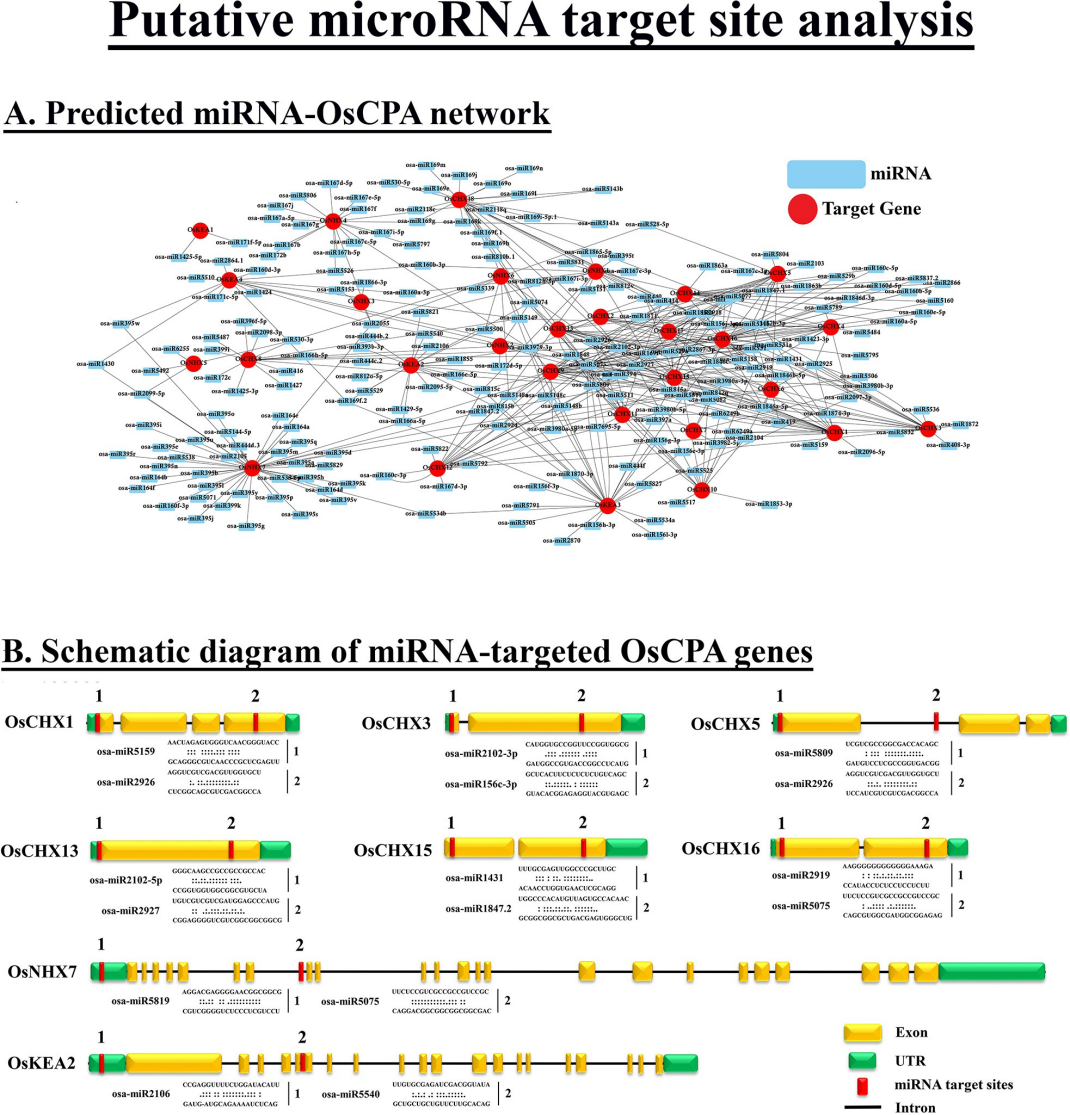
Predicted miRNAs target site analysis. **A.** The network illustrates the predicted miRNA targeting *OsCPA* genes. Light blue rectangles represent the putative miRNAs and red circles represent the targeted *OsCPA* genes. **B.** The schematic diagram represents the *OsCPA* genes targeted by miRNAs and the red color represents the putative miRNA target sites of each gene.

miRNAs are small noncoding sequences, involved in post-transcriptional regulations of gene expression through translation or cleaving of target mRNA. The regulatory functions of miRNA have previously been demonstrated in various plant species [75, 76]. To elucidate the roles of miRNAs in *OsCPA* gene regulations, 124 miRNA families targeting 29 *OsCPA* genes were retrieved and illustrated as a network (Fig 10A and 10B and S9 Data). The number of putative miRNA target sites for each gene ranged from 2 (*OsKEA1*) to 43 (*OsNHX7*), with a length of 19 to 24 nucleotides. Based on this study, osa-miR531 had the highest number of reads (27), followed by osa-miR395 (23), osa-miR1858 (18), and osa-miR160 (18) (Table 2). The most abundant miRNA family, osa-miR531, targeted only 6 *OsCHXs* including *OsCHX2*, *OsCHX3*, *OsCHX4*, *OsCHX5*, *OsCHX6*, and *OsCHX9* with *OsCHX9* being the most frequently targeted gene, suggesting their involvement in signal transduction pathways, innate immunity, drought response, biomass enhancement, and metabolism pathways [77–79]. The second largest miRNA family, osa-miR395 targeted only *OsKEA4*, *OsNHX1*, and *OsNHX7* with *OsNHX7* being a highly targeted gene. The osa-miR395 family was predicted to be involved in sulfate assimilation and increased sulfate translocation from roots to shoots [80, 81]. The osa-miR1858 family was predicted to target 7 *OsCPA* genes, namely *OsNHX1*, *OsCHX4*, *OsCHX13*, *OsCHX15*, *OsCHX16*, *OsCHX17*, and *OsCHX18* with *OsCHX13*, and *OsCHX15* being mostly targeted, thereby regulating the post-transcription modification of S-adenosyl-methionine genes in rice [82]. Furthermore, osa-miR160 targeted 6 *OsCPA* genes such as *OsKEA4*, *OsNHX1*, *OsNHX7*, *OsCHX12*, *OsCHX14*, and *OsCHX16* with *OsCHX14*, and *OsCHX16* being highly targeted genes. MiR160 plays potential roles in seed germination, floral organ development, *in vitro* shoot regeneration, root cap formation, and regulations of auxin signaling pathways [83–85]. Among all *OsCPA* genes, *OsNHX7* was the most targeted, being targeted by 43 miRNAs followed by *OsKEA3*, and *OsCHX9*, targeted by 27 and 25 miRNAs, respectively. This study suggests that *OsCPA* genes are potentially involved in numerous biological functions and plant developmental processes.

**Table 2:** Information about abundant miRNA ID, functions, and their targeted *OsCPA* genes.

miRNA ID	Functions	Targeted genes	References
osa-miR531	Involved in signal transduction pathways, innate immunity, drought response, biomass enhancement, and metabolism pathways.	*OsCHX2*, *OsCHX3*, *OsCHX4*, *OsCHX5*, *OsCHX6*, *OsCHX9*	[77–79]
osa-miR395	Involved in sulfate assimilation and increased sulfate translocation from roots to shoots.	*OsKEA4*, *OsNHX1*, *OsNHX7*	[80, 81]
osa-miR1858	Regulate the post-transcription modification of S-adenosyl-methionine genes in rice.	*OsNHX1*, *OsCHX4*, *OsCHX13*, *OsCHX15*, *OsCHX16*, *OsCHX17*, *OsCHX18*	[82]
osa-miR160	Involved in seed germination, floral organ development, *in vitro* shoot regeneration, root cap formation, and regulations of auxin signaling pathways.	*OsKEA4*, *OsNHX1*, *OsNHX7*, *OsCHX12*, *OsCHX14*, *OsCHX16*	[83–85]

### 3.12 Gene ontology (GO) analysis of *OsCPA* genes

**Fig 11:**
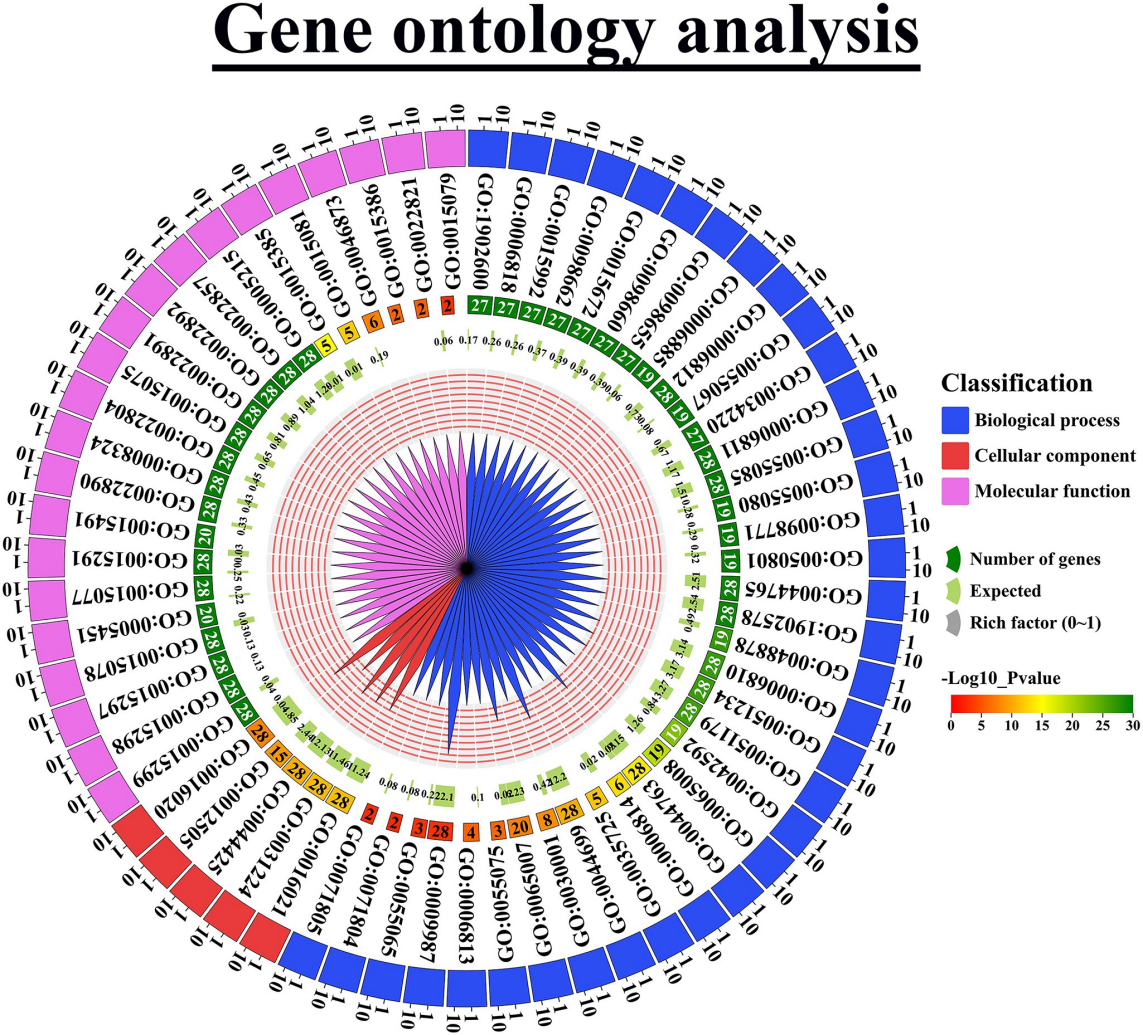
GO analysis of *OsCPA* genes. GO enrichment analysis of differentially expressed genes (DEGs) is represented by a diagram. GO terms of biological functions (biological process, cellular component, and molecular functions) of these DEGs are shown on the right side of the circle and the number of the corresponding *OsCPA* gene is shown inside of the circle by different colors.

GO analysis was conducted to elucidate the potential functions of *OsCPA* genes. It provides a framework for classifying genes into three major categories based on their functions, including biological process (57.14%), cellular component (7.94%), and molecular function (34.92%) (Fig 11 and S10 Data). The biological process categories included 36 subcategories and the major subcategories contained 28 *OsCPA* genes (96.55%) including transport (GO:0006810; *p*-value: 2.10E-27), localization (GO:0051179; *p*-value: 6.80E-27), single-organism process (GO:0044699; *p*-value: 7.70E-11), and cellular process (GO:0009987; *p*-value: 0.0013). In cellular component categories, 4 subcategories were highly represented (96.55% genes), including integral component of membrane (GO:0016021; *p*-value: 7.70E-12), intrinsic component of membrane (GO:0031224; *p*-value: 1.30E-11), membrane part (GO:0044425; *p*-value: 6.50E-11), and membrane (GO:0016020; *p*-value: 1.90E-08). In molecular functions categories, maximum representation (96.55% genes) were observed in antiporter activity (GO:0015297; *p*-value: 1.00E-30), transporter activity (GO:0005215; *p*-value: 1.00E-30), trans-membrane transporter activity (GO:0022857; *p*-value: 1.00E-30), substrate-specific transporter activity (GO:0022892; *p*-value: 1.00E-30), active transmembrane transporter activity (GO:0022804; *p*-value: 1.00E-30), and ion transmembrane transporter activity (GO:0015075; *p*-value: 1.00E-30). The major roles of *OsCPA* genes were predicted to involve ion transportation, and protein localization on membrane parts. These findings are supported by several studies that speculated the various biological, molecular, and cellular activities of *CPA* genes [86]. This study revealed that the OsCPA gene family members were distributed across potential GO categories.

### 3.13 Regulatory relationship between transcription factors (TFs) and Os*CPA* genes

**Fig 12:**
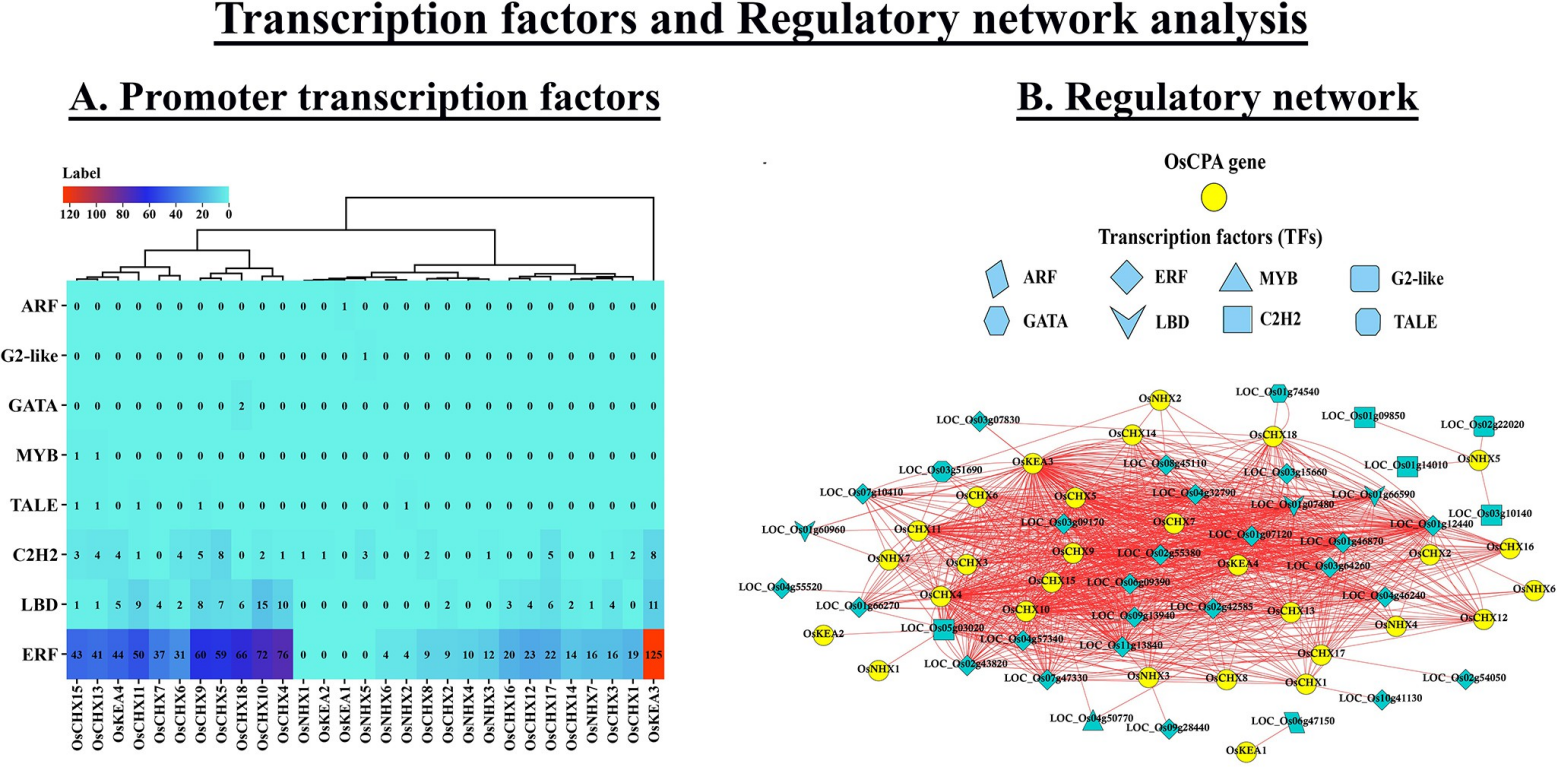
TFs analysis of *OsCPA* genes. **A.** A heatmap represents the predicted TFs in *OsCPA* gene promoters. The name of each *OsCPA* gene is aligned on the bottom of the heatmap. The major eight TFFs are shown on the left side of the heatmap. The color intensity was also shown on top of the heatmap. **B.** The regulatory network between TFs and *OsCPA* genes. The interactions between *OsCPA* genes and their regulatory TFs are represented through the regulatory network. The yellow circles indicate *OsCPA* genes and the light blue color shapes represent TF families.

TFs are proteins involved in the transcriptional regulations of genes in response to stress, defense, and developmental processes. In this study, a total of 40 TFs distributed among 12 TFFs were identified in the promoter region of *OsCPA* genes (Fig 12A and 12B and S11 Data). Among all TFFs, ERF, C2H2, LBD, TALE, GATA, MYB, G2-like, and ARF were selected as main TFFs accounting for 90% (36TFs) of all identified TFFs. The connections between TFFs and the candidate *OsCPA* genes were demonstrated through network and sub-network analysis. The ERF family, containing 24 TFs and 849 of 1022 transcription factor binding sites (TFBS) showed a strong connection with 25 *OsCPA* genes. ERF was frequently identified in the promoter region of *OsKEA3*, *OsCHX4*, and *OsCHX10*. Similarly, LBD, C2H2, TALE, MYB, GATA, G2-like, and ARF were linked to 19, 18, 5, 2, 1, 1, and 1 *OsCPA* genes, respectively. The maximum numbers of TFFs (5TFFs) were associated with *OsCHX13*, and *OsCHX15* followed by *OsCHX9*, and *OsCHX11* with 4 TFFs.

The ERF is one of the largest TF families exhibiting potential functions in the transcriptional regulation of plant developmental processes such as flower development, embryo development, and spikelet meristem determinacy [87, 88]. They also respond to various environmental anomalies, including salinity, cold, and drought stresses. Moreover, ERFs participate in various gene regulations by binding to *cis*-elements of target genes [89]. Thus, it is speculated that *OsKEA3*, *OsCHX4*, and *OsCHX10* might be significantly involved in plant development. The C2H2, a zinc finger TF family regulates isoflavone accumulation and responds to various environmental stimuli [90]. The GATA zinc finger DNA binding factor either activates or represses the transcription processes of genes to control the development of diverse tissues [91]. The LBD family is involved in the lateral organ developmental process, morphogenesis mechanisms, and metabolism regulation [92]. The TALE is also an important TFF that regulates the signal transduction process, meristem formation, as well as organ morphogenesis in plants [93]. The MYB family has important functions in cell identity, flower development, stress responses, defense mechanisms, and metabolism in plants [94]. The ARF family binds to auxin response elements (AuxREs) in gene promoters and regulates auxin-related gene expression. Thus, the relationship between *OsCPA* genes and TFs demonstrates the diverse expression profiles of *OsCPA* genes in controlling plant physiological growth, and cellular development.

### 3.14 Protein-protein interaction (PPI) network prediction of OsCPA proteins

**Fig 13:**
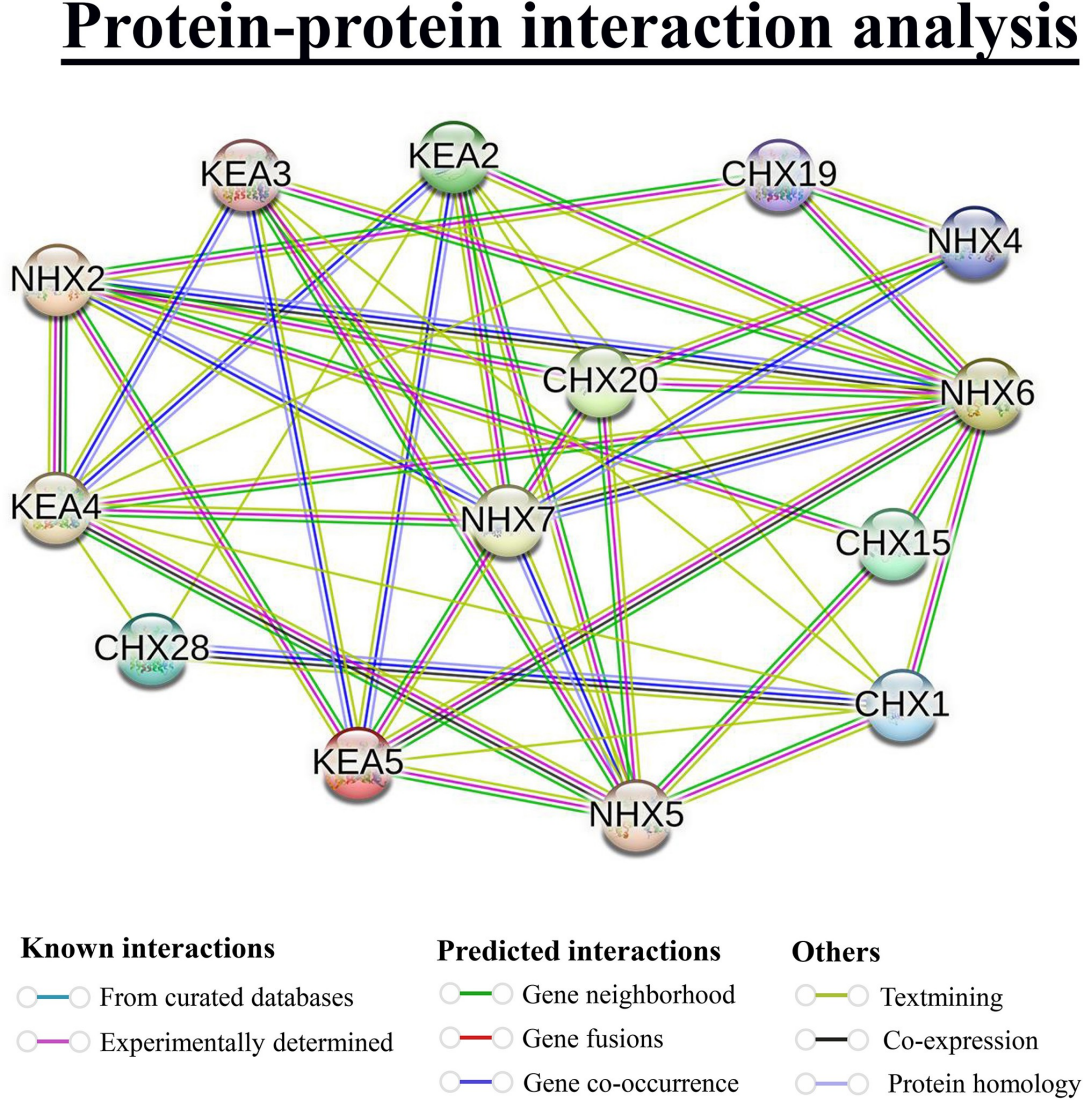
The PPI network of OsCPA proteins. The proteins are represented as network nodes and the colored lines indicate different data sources. Thicker lines represent a higher coefficient.

A PPI network of OsCPA proteins was constructed using STRING online tools based on *Arabidopsis* orthologs to illustrate the potential regulatory functions of CPA proteins in rice (Fig 13 and S12 Data). The OsCPA proteins exhibited strong homology with *Arabidopsis* STRING proteins. In total, 11 OsCPA proteins were found to be homologous to *Arabidopsis* CHX15 and interacted with NHX2, NHX5, and NHX6, suggesting their involvement in pollen development [95]. Furthermore, OsCHX4, OsCHX6, and OsCHX17; OsCHX5, and OsCHX7 proteins were homologous with *Arabidopsis* CHX19, and CHX20, which interacted strongly with NHX2, NHX4, NHX6, and KEA4; NHX2, NHX4, NHX5, NHX6, KEA2, and KEA5, respectively. Additionally, AtCHX19 and AtCHX20 are involved in K^+^ and pH homeostasis within dynamic endomembrane [96]. OsNHX1, OsNHX3, and OsNHX6 were also homologous to *Arabidopsis* STRING protein NHX2 and showed interaction with KEA4, KEA5, NHX6, NHX7, CHX15, CHX19, and CHX20. AtNHX regulates pH and K^+^ concentrations in plant cells which is crucial for flower development, pollen wall formation, embryo development, and cell expansion [97]. OsKEA1, OsKEA2, OsKEA3, OsKEA4, OsNHX2, OsNHX4, OsNHX5, OsNHX7, OsCHX11, and OsCHX18 were homologous with KEA5, KEA2, KEA4, KEA3, NHX4, NHX6, NHX5, NHX7, CHX1, and CHX28, respectively. KEAs are expressed in both the shoot and root of *Arabidopsis*. The expression of KEA2-4 was enhanced under low K^+^ stress, while KEA2 and KEA5 were induced by sorbitol and ABA treatments. These proteins may play roles in K^+^ homeostasis, osmotic adjustment, and regulations of photosynthesis in plants [98]. Additionally, NHX4-6 enhances the salt tolerance and auxin distribution in plants [99]. Therefore, the interacted CPA proteins in rice and *Arabidopsis* might have similar functional patterns in signal transduction, ion homeostasis, organ development, and hormonal responses.

### 3.15 Expression pattern analysis of Os*CPA* genes in different tissues

**Fig 14:**
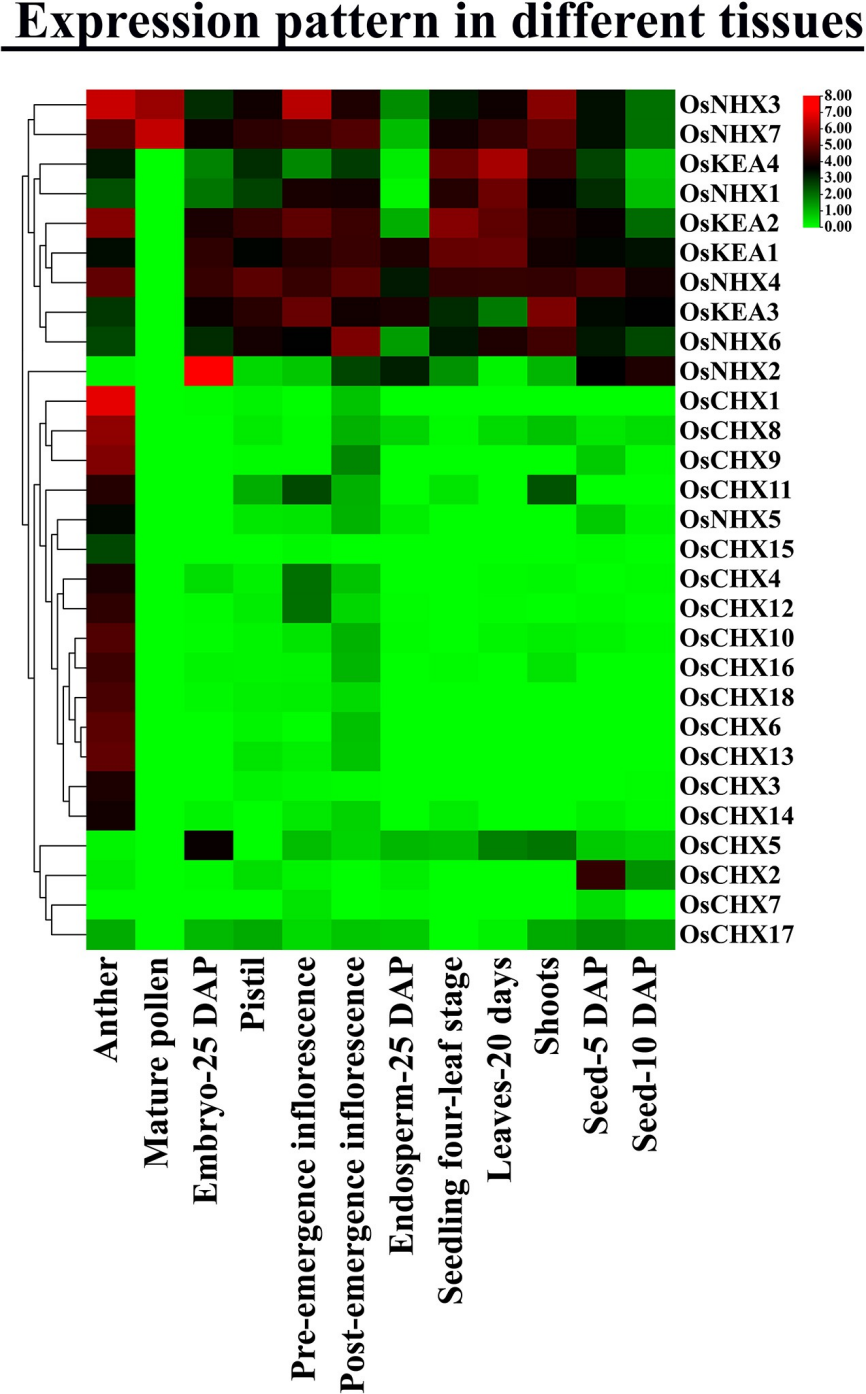
Expression profiles of *OsCPA* genes in 12 tissues. The name of each *OsCPA* gene is shown on the right side and the tissue types are represented at the bottom of the heatmap. The expression values were mapped using a color gradient from green to red (count = 0–8) shown on the right side of the heatmap. The abbreviation “DAP” on the tissue label represents “Days after pollination”.

To reveal the putative expressional profiles of *OsCPA* genes, RNA-seq data were utilized to investigate their expression in 12 different tissues of *O*. *sativa* at various developmental stages, including anther, mature pollen, embryo-25 DAP (days after pollination), pistil, pre-emergence inflorescence, post-emergence inflorescence, endosperm- 25 DAP, seedling four-leaf stage, leaves-20 days, shoots, seed-5 DAP, and seed-10 DAP (Fig 14 and S13 Data). Each *OsCPA* gene was expressed in at least one tissue. *OsCHX1*, *OsNHX7*, *OsNHX2*, *OsNHX4*, *OsNHX3*, *OsNHX6*, *OsKEA1*, *OsKEA2*, *OsKEA4*, *OsNHX3*, *OsNHX4*, and *OsNHX2* exhibited a higher level of expression in aforementioned tissues, respectively. Meanwhile, all *OsCPA* genes were expressed in pre-emergence inflorescence tissue and most of all *OsCPA* genes exhibited higher expression in anther, suggesting their involvement in various developmental stages of reproductive organs. However, in mature pollen, *OsNHX3* and *OsNHX7* were significantly expressed, indicating their roles in reproductive organ development.

Among the *OsKEA* group, *OsKEA1*, and *OsKEA2* genes exhibited elevated expression in most tissues, particularly in leaves-20 days and seedling four-leaf stage, respectively. The *OsKEA3* is potentially expressed in shoots and *OsKEA4* was recommended as a highly expressed gene in leaves-20 days suggesting the potentiality of *OsKEAs* in tissue development. The involvement of *AtKEA1*-*AtKEA3*, the orthologs of *OsKEA2*, and *OsKEA4* in photosynthesis and chloroplast osmoregulation suggested a similar functional pattern for them [100, 101].

In the case of *OsNHXs*, *OsNHX3* and *OsNHX7* were dominantly expressed in all tissues. *OsNHX3*, *OsNHX4*, and *OsNHX5* genes were predominantly expressed in anther, while *OsNHX1*, *OsNHX2*, *OsNHX6*, and *OsNHX7* were prominently expressed in leaves-20 days, embryo-25 DAP, post-emergence inflorescence, and mature pollen, respectively. The *AtNHX3* and *AtNHX4* genes, the orthologs of *OsNHX*2 and *OsNHX3* have essential roles in seed development [102, 103]. Similar expression patterns of the NHX group have been observed in other species. For instance, in wheat *TaNHX2*, *TaNHX4*, *TaNHX6*, and *TaNHX12* showed significant expression in grain development. Additionally, *TaNHX7*, *TaNHX8*, and *TaNHX11* were highly expressed in leaf and spike at various developmental stages, suggesting their crucial roles in growth and development [40]. Thus, the *OsNHX* genes are mostly involved in the reproduction process and tissue development.

The *OsCHX* genes showed a lower level of expression in all tissues except the anther, and *OsCHX1* was highly expressed in the anther, suggesting their potential role in reproduction. The CHX group genes have the potential for developing male sterility lines or modifying fertility in crops, as well [104]. The expression of *AtCHX15*, an ortholog of *OsCHX1* was observed in pollen, demonstrating its involvement in pollen grain development for successful reproduction [105]. In wheat, *TaCHX* genes displayed higher expression in the spike, also suggesting their functions in reproductive organ development [40]. Notably, *OsCHX7* was expressed only in pre-emergence inflorescence, seed-5 DAP, and endosperm-25 DAP, which might indicate its specific role in reproduction. Similarly, *GmNHX3* in soybean was expressed only in seed, demonstrating particular roles in seed development [58]. Therefore, OsKEA and OsNHX groups are highly involved in rice tissue development rather than OsCHX and *OsCPA* genes including *OsKEA1*, *OsKEA2*, *OsNHX3*, and *OsNHX7* are considered candidates for tissue-specific expression.

### 3.16 Transcriptomic expression analysis of *OsCPA* genes in response to phytohormones

**Fig 15:**
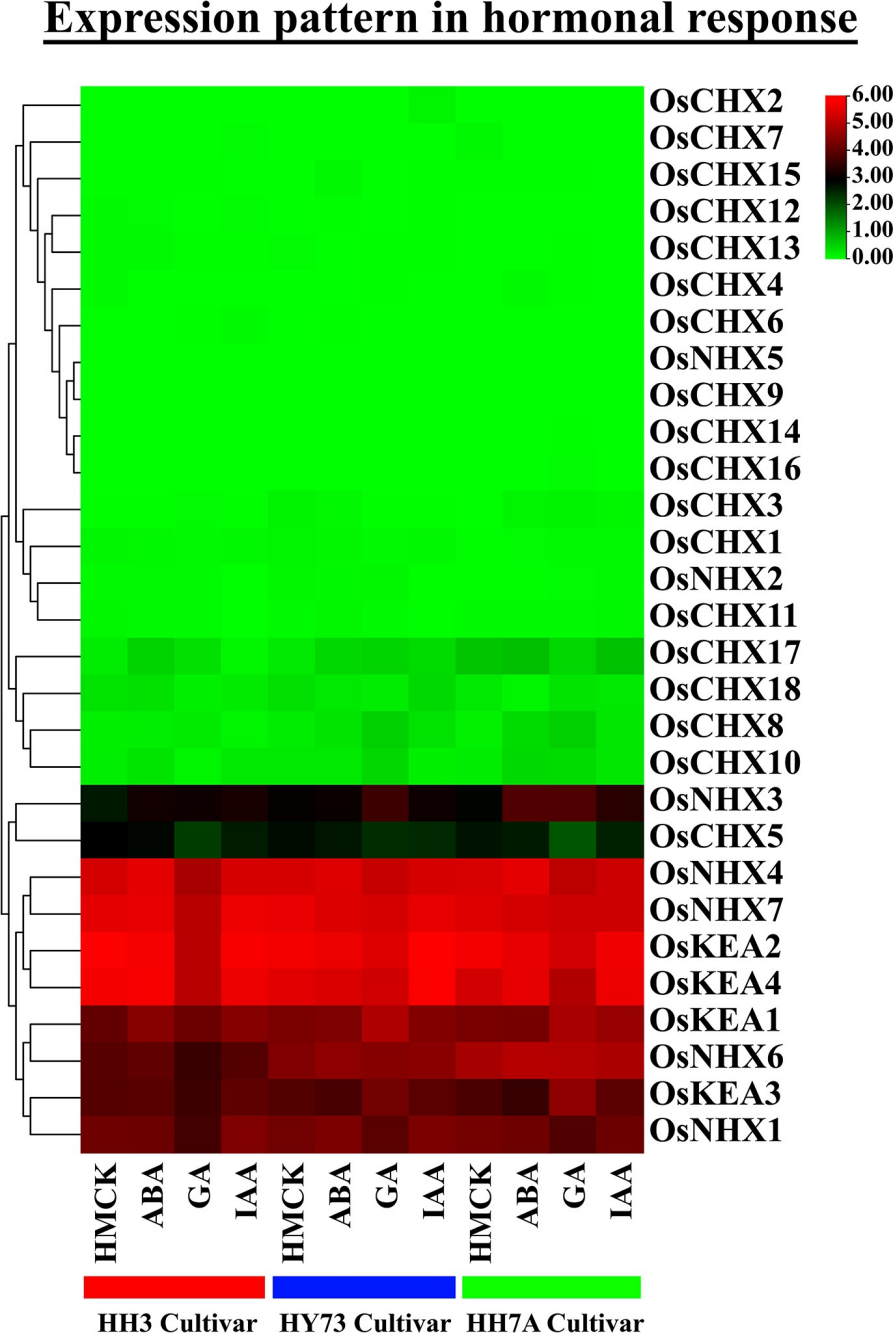
Expression profiles of *OsCPA* genes under hormonal treatments. The name of each *OsCPA* gene is shown on the right side of the heatmap and the name of the rice varieties and hormones are represented at the bottom of the heatmap. The red, blue, and green colors represent HH3, HY73, and HH7A varieties, respectively. The expression values were mapped using a color gradient from green to red (count = 0–6) shown on the right side of the heatmap.

The relative expression profiles of 29 *OsCPA* genes in 15 days of leaf tissues of three different rice varieties including HH3, HY73, and HH7A were studied in response to ABA, GA, and IAA with control HMCK (Fig 15 and S14 Data). The number of DEGs varied according to varieties and hormonal treatment. *OsNHX3* was up-regulated in all three varieties in response to ABA, GA, and IAA while *OsNHX5* and *OsCHX9* were down-regulated. Interestingly, in HH3 and HY73 varieties, down-regulations occurred slightly higher than up-regulations while in HH7A the number of down-regulated genes and up-regulated genes were equal (6 *OsCPAs*) across all three hormonal treatments. The analyzed three varieties were enriched with the elevated expression patterns of *OsKEA1*, and *NHX3*; *OsKEA1*, *OsNHX3*, *OsNHX6*, *OsCHX8*; *OsNHX3*, *OsNHX6*, *OsCHX1*, *OsCHX3*, *OsCHX8*, and *OsCHX10*, respectively. It can be speculated that the *OsCPA* genes are most induced in the HH7A variety.

Regarding hormonal treatment, 11 *OsCPAs* were up-regulated in HH3, 12 *OsCPAs* in HY73, and 10 *OsCPAs* in HH7A in response to ABA hormone. Moreover, *OsNHX3*, *OsNHX4*, *OsNHX6*, *OsNHX7*, *OsCHX8*, and *OsCHX17* were induced in all three varieties in response to ABA treatment. The CAREs have shown the presence of ABRE elements in the promoters of ABA-responsive genes. Typically, ABA is a signaling molecule for seed dormancy, but involved in plant defense mechanisms in environmental anomalies such as drought, salinity, cold, and heat stresses that trigger to enhance ABA levels [106, 107]. It suggests that these *OsCPA* genes exhibit potential roles in plant seed development and adaptation to environmental stresses. In GA hormonal treatment, 7, 8, and 14 *OsCPA* genes exhibited higher expression patterns compared to control. Additionally, *OsKEA1*, *OsNHX3*, and *OsCHX8* exhibited elevated expression patterns in all varieties in response to GA treatment. GA hormone was first isolated from *Gibberella fujikuroi*- a rice pathogen, and identified as a growth regulator that enhances rice root growth [108]. It is observed that ABA is responsible for seed dormancy, while GA promotes seed germination. Thus, both ABA and GA regulate the expression of genes involved in seed and flower development [109]. Furthermore, a total of 9, 13, and 12 *OsCPA* genes were rapidly induced in response to IAA treatment. *OsKEA1*, *OsKEA3*, *OsNHX1*, and *OsNHX3* were upregulated in the aforementioned varieties in IAA treatment. IAA interacts with ARF transcription factors to enhance auxin responses [110]. In summary, maximum *OsCPA* genes were up-regulated in the HH7A variety, and *OsNHX3* was demonstrated as a candidate gene for all treatments. Moreover, *OsNHX3*, *OsNHX4*, *OsNHX6*, *OsNHX7*, *OsCHX8*, and *OsCHX17*; *OsKEA1*, *OsNHX3*, and *OsCHX8*; *OsKEA1*, *OsKEA3*, *OsNHX1*, and *OsNHX3* were potential candidate for ABA, GA, and IAA hormonal treatment, respectively.

## 4.0 Conclusion

In this study, 29 *OsCPA* genes were identified and characterized at the genome level for the first time using comprehensive bioinformatics analysis. The *OsCPA* genes had a conserved structural nature, however, significant differences were observed from the members of other groups. The selection pressure analysis, collinear, and synteny analysis gain insight into the functional stability as well as the evolution of *OsCPA* genes. The predicted CAREs might have potential roles in the transcriptional levels of *OsCPA* genes. GO analysis indicated the involvement of the majority of *OsCPA* genes in various biological processes, such as ion transportation and ion homeostasis. Moreover, the presence of TFs and miRNA molecules in gene promoter regions suggests their potential functions in signal transduction, plant organ development, immunity response, metabolism, hormonal response, and stress responses. The PPI analysis predicted the higher homogeneity between rice and *Arabidopsis* CPA proteins. The tissue-specific RNA-seq data showed elevated expression of most *OsCPA* genes in anther. *OsNHX3*, *OsNHX7*, *OsKEA1*, and *OsKEA2* were identified as candidate genes, showing induced expression profiles in rice tissues. Furthermore, the expression profiles of *OsCPA* genes varied across varieties and hormones. Maximum numbers of genes were induced in HH7A varieties, while *OsCPA* genes are highly responsive to ABA hormones and *OsNHX3* was up-regulated in all varieties in response to all hormonal treatments. Our findings provide valuable insights for future functional characterization of *CPA* genes in plant developmental processes related to agronomically important traits.

## Supporting information

S1 Data Full-length protein sequences of CPA gene families of *A*. *thaliana* and *O*. *sativa* for constructing a phylogenetic tree. (TXT)

S2 Data Full-length coding sequences of OsCPA gene family members of *O*. *sativa* plant species. (TXT)

S3 Data Full-length genomic sequences of OsCPA gene family members of *O*. *sativa* plant species. (TXT)

S4 Data Full-length protein sequences of OsCPA gene family members of *O*. *sativa* plant species. (TXT)

S5 Data *In silico* predicted number of introns and exons in *OsCPA* genes. (DOCX)

S6 Data Time of gene duplication estimated for different paralogous pairs of *OsCPA* genes based on Ka and Ks values. (XLSX)

S7 Data The promoter region (2.0 kb genomic sequences) of OsCPA gene family members of *O*. *sativa* for analysis of *cis*-acting regulatory elements. (TXT)

S8 Data The predicted *cis*-acting regulatory elements of the 5’ untranslated regions (UTRs) (2.0 kb genomic sequences) of *OsCPA* genes of *O*. *sativa*. (XLSX)

S9 Data miRNA targeted prediction of *OsCPAs*. The miRNA data was downloaded from the plant micro-RNA encyclopedia (http://pmiren.com/).
(DOCX)

S10 Data The details GO analysis of the predicted *OsCPA* genes was performed using the Plant Transcription Factor Database (Plant TFDB, http://planttfdb.cbi.pku.edu.cn//). (XLSX)

S11 Data Identified the main 8 TF families associated with the regulation of identified *OsCPA* genes in the *O*. *sativa* genome. (XLSX)

S12 Data Protein-protein interaction of OsCPA proteins with *Arabidopsis* string proteins retrieved from STRING database. (XLSX)

S13 Data Tissue-specific expression profiles of *OsCPA* genes. (XLSX)

S14 Data RNA-seq expression data of *OsCPA* genes in phytohormone treatments. (XLSX)
