# Supplementary material for: Genome-wide identification and characterization of cation-proton antiporter (CPA) gene family in rice (Oryza sativa L.) and their expression profiles in response to phytohormones
Source: PLoS One. 2025 Jan 24;20(1):e0317008. doi: 10.1371/journal.pone.0317008 (PMC11761165; doi:10.1371/journal.pone.0317008)
Supplement: S5 Data — (DOCX) [file pone.0317008.s005.docx]

**S5 Data. *In silico* predicted number of introns and exons in *OsCPA* genes.**

| **Group** | **Gene name** | **Intron** | **Exon** |
| --- | --- | --- | --- |
| **CHX** | *OsCHX1* | 3 | 4 |
|  | *OsCHX2* | 0 | 1 |
|  | *OsCHX3* | 1 | 2 |
|  | *OsCHX4* | 1 | 2 |
|  | *OsCHX5* | 2 | 3 |
|  | *OsCHX6* | 1 | 2 |
|  | *OsCHX7* | 2 | 3 |
|  | *OsCHX8* | 2 | 3 |
|  | *OsCHX9* | 1 | 2 |
|  | *OsCHX10* | 1 | 2 |
|  | *OsCHX11* | 3 | 4 |
|  | *OsCHX12* | 1 | 2 |
|  | *OsCHX13* | 0 | 1 |
|  | *OsCHX14* | 13 | 14 |
|  | *OsCHX15* | 1 | 2 |
|  | *OsCHX16* | 1 | 2 |
|  | *OsCHX17* | 1 | 2 |
|  | *OsCHX18* | 0 | 1 |
| **KEA** | *OsKEA1* | 8 | 9 |
|  | *OsKEA2* | 19 | 20 |
|  | *OsKEA3* | 19 | 20 |
|  | *OsKEA4* | 17 | 18 |
| **NHX** | *OsNHX1* | 13 | 14 |
|  | *OsNHX2* | 12 | 13 |
|  | *OsNHX3* | 13 | 14 |
|  | *OsNHX4* | 17 | 18 |
|  | *OsNHX5* | 10 | 11 |
|  | *OsNHX6* | 12 | 13 |
|  | *OsNHX7* | 22 | 23 |
