# Supplementary material for: Genome-wide identification and characterization of cation-proton antiporter (CPA) gene family in rice (Oryza sativa L.) and their expression profiles in response to phytohormones
Source: PLoS One. 2025 Jan 24;20(1):e0317008. doi: 10.1371/journal.pone.0317008 (PMC11761165; doi:10.1371/journal.pone.0317008)
Supplement: S9 Data — The miRNA data was downloaded from the plant micro-RNA encyclopedia (http://pmiren.com/). (DOCX) [file pone.0317008.s009.docx]

**S9 Data. miRNA targeted prediction of *OsCPAs*. The miRNA data was downloaded from the plant micro RNA**

**encyclopedia (http://pmiren.com/). (Doc)**

| **miRNA ID** | **Targeted gene** | **miRNA length** | **Target start** | **Target end** | **miRNA_aligned_fragment** |
| --- | --- | --- | --- | --- | --- |
| osa-miR2925 | *OsCHX3* | 19 | 721 | 739 | UGGCGGCCGCGGGCUUCGU |
| osa-miR5534a | *OsKEA3* | 21 | 1532 | 1552 | UGACGACAACAGCUAGAAUGG |
| osa-miR6249a | *OsCHX1* | 21 | 766 | 786 | CGUGAAGAGCUCGCCGGCGGC |
| osa-miR6249a | *OsCHX10* | 21 | 292 | 312 | CGUGAAGAGCUCGCCGGCGGC |
| osa-miR6249b | *OsCHX1* | 21 | 766 | 786 | CGUGAAGAGCUCGCCGGCGGC |
| osa-miR6249b | *OsCHX10* | 21 | 292 | 312 | CGUGAAGAGCUCGCCGGCGGC |
| osa-miR5075 | *OsNHX7* | 21 | 3299 | 3319 | UUCUCCGUCGCCGCCGUCCGC |
| osa-miR5075 | *OsCHX4* | 21 | 1516 | 1536 | UUCUCCGUCGCCGCCGUCCGC |
| osa-miR1430 | *OsNHX7* | 21 | 1733 | 1753 | UGGUGAGCCUUCCUGGCUAAG |
| osa-miR1846d-3p | *OsCHX16* | 20 | 1911 | 1930 | UAUCCGGCGCCGCAGGGAGG |
| osa-miR1846d-3p | *OsCHX14* | 20 | 1860 | 1879 | UAUCCGGCGCCGCAGGGAGG |
| osa-miR2919 | *OsCHX9* | 19 | 191 | 209 | AAGGGGGGGGGGGGAAAGA |
| osa-miR2927 | *OsCHX4* | 23 | 1608 | 1630 | UGUCGUCGUCGAUGGAGCCCAUG |
| osa-miR414 | *OsCHX18* | 21 | 1256 | 1276 | UCAUCCUCAUCAUCAUCGUCC |
| osa-miR5075 | *OsCHX9* | 21 | 21 | 41 | UUCUCCGUCGCCGCCGUCCGC |
| osa-miR5075 | *OsCHX12* | 21 | 2092 | 2111 | UUCUCCGUCGCCGCCGUCCGC |
| osa-miR5153 | *OsNHX3* | 24 | 50 | 73 | UGGAUUCCACUGACACGUAGACGU |
| osa-miR530-3p | *OsCHX8* | 21 | 1838 | 1858 | AGGUGCAGAGGCAGAUGCAAC |
| osa-miR5484 | *OsCHX4* | 21 | 1784 | 1804 | AACCGAGCGCGCUGUUGAUUA |
| osa-miR810b.1 | *OsNHX6* | 21 | 215 | 235 | UGAACACCGAUAUGCGUCAUC |
| osa-miR1424 | *OsKEA4* | 21 | 78 | 98 | AUGCACACUGAUGCUGAUUGU |
| osa-miR1437b-3p | *OsCHX16* | 24 | 609 | 632 | GUGCUGGCGAGCUCCGGUGCCGCA |
| osa-miR156j-3p | *OsCHX4* | 22 | 1381 | 1402 | GCUCGCUCCUCUUUCUGUCAGC |
| osa-miR160a-3p | *OsKEA4* | 21 | 360 | 380 | GCGUGCAAGGAGCCAAGCAUG |
| osa-miR160b-3p | *OsKEA4* | 21 | 360 | 380 | GCGUGCAAGGAGCCAAGCAUG |
| osa-miR1847.2 | *OsCHX15* | 24 | 2215 | 2238 | UGGCCCACAUGUUAGUGCCACAAC |
| osa-miR1848 | *OsKEA4* | 21 | 226 | 246 | CCUCGCCGGCGCGCGCGUGCA |
| osa-miR1848 | *OsCHX10* | 21 | 285 | 304 | CCUCGCCGGCGCGCGCGUGCA |
| osa-miR1848 | *OsCHX5* | 21 | 2374 | 2394 | CCUCGCCGGCGCGCGCGUGCA |
| osa-miR1858a | *OsCHX15* | 21 | 261 | 281 | GAGAGGAGGACGGAGUGGGGC |
| osa-miR1858a | *OsCHX13* | 21 | 261 | 281 | GAGAGGAGGACGGAGUGGGGC |
| osa-miR1858b | *OsCHX13* | 21 | 261 | 281 | GAGAGGAGGACGGAGUGGGGC |
| osa-miR1858b | *OsCHX15* | 21 | 261 | 281 | GAGAGGAGGACGGAGUGGGGC |
| osa-miR2055 | *OsNHX3* | 21 | 113 | 133 | UUUCCUUGGGAAGGUGGUUUC |
| osa-miR2055 | *OsNHX2* | 21 | 122 | 142 | UUUCCUUGGGAAGGUGGUUUC |
| osa-miR2055 | *OsNHX6* | 21 | 110 | 130 | UUUCCUUGGGAAGGUGGUUUC |
| osa-miR2096-5p | *OsCHX1* | 21 | 839 | 859 | UGCCGAUUUCCCCCUCGGGCG |
| osa-miR2097-3p | *OsCHX4* | 22 | 1378 | 1399 | UUCUCUUCUUCGUGUCGCAUUU |
| osa-miR2864.1 | *OsKEA4* | 21 | 1462 | 1482 | UUUUGCUGCCCUUGUUUUGCA |
| osa-miR2925 | *OsCHX1* | 19 | 2470 | 2488 | UGGCGGCCGCGGGCUUCGU |
| osa-miR2926 | *OsCHX14* | 20 | 577 | 596 | AGGUCGUCGACGUUGGUGCU |
| osa-miR2926 | *OsCHX16* | 20 | 628 | 647 | AGGUCGUCGACGUUGGUGCU |
| osa-miR395a | *OsNHX7* | 21 | 1939 | 1959 | GUGAAGUGCUUGGGGGAACUC |
| osa-miR395u | *OsNHX7* | 21 | 1939 | 1959 | GUGAAGCGUUUGGGGGAAAUC |
| osa-miR3979-3p | *OsKEA3* | 20 | 1183 | 1202 | CUUCGGGGGAGGAGAGAAGC |
| osa-miR3980a-5p | *OsCHX7* | 21 | 187 | 207 | AAUCGACGGCCUCAGUCAGGG |
| osa-miR3980b-5p | *OsCHX7* | 21 | 187 | 207 | AAUCGACGGCCUCAGUCAGGG |
| osa-miR3982-5p | *OsCHX7* | 21 | 237 | 257 | GCGCUCCACGUAGGCAACAAU |
| osa-miR444d.3 | *OsNHX7* | 21 | 1077 | 1096 | UUGUGGCUUUCUUGCAAGUUG |
| osa-miR5075 | *OsCHX14* | 21 | 924 | 944 | UUCUCCGUCGCCGCCGUCCGC |
| osa-miR5075 | *OsCHX16* | 21 | 975 | 995 | UUCUCCGUCGCCGCCGUCCGC |
| osa-miR5082 | *OsCHX7* | 24 | 352 | 375 | UGCGAUGAUGGCCGCGCGGGUUCA |
| osa-miR5151 | *OsNHX1* | 22 | 700 | 721 | UAAUGAUGUGGGUACGAAUGAA |
| osa-miR530-5p | *OsCHX18* | 20 | 2116 | 2135 | UGCAUUUGCACCUGCACCUA |
| osa-miR531a | *OsCHX9* | 24 | 631 | 654 | CUCGCCGGGGCUGCGUGCCGCCAU |
| osa-miR531a | *OsCHX6* | 24 | 523 | 546 | CUCGCCGGGGCUGCGUGCCGCCAU |
| osa-miR531b | *OsCHX9* | 20 | 635 | 654 | CUCGCCGGGGCUGCGUGCCG |
| osa-miR531b | *OsCHX6* | 20 | 527 | 546 | CUCGCCGGGGCUGCGUGCCG |
| osa-miR531c | *OsCHX9* | 24 | 631 | 654 | CUCGCCGGGGCUGCGUGCCGCCAU |
| osa-miR531c | *OsCHX6* | 24 | 523 | 546 | CUCGCCGGGGCUGCGUGCCGCCAU |
| osa-miR5517 | *OsCHX10* | 21 | 1722 | 1742 | UGCAUCACGGCGCAUAUGUAG |
| osa-miR5534b | *OsKEA3* | 21 | 1532 | 1552 | CGAUGACAACAGCUAGAAUGG |
| osa-miR5534b | *OsNHX7* | 21 | 1022 | 1042 | CGAUGACAACAGCUAGAAUGG |
| osa-miR5809 | *OsCHX5* | 20 | 6 | 25 | UCGUCGCCGGCGACCACAGC |
| osa-miR5809 | *OsCHX10* | 20 | 106 | 125 | UCGUCGCCGGCGACCACAGC |
| osa-miR5819 | *OsNHX7* | 21 | 108 | 128 | AGGACGAGGGGAACGGCGGCG |
| osa-miR6255 | *OsNHX5* | 22 | 329 | 350 | UGGGAAAAAUGGGCAGUUGAGU |
| osa-miR812q | *OsCHX1* | 24 | 1234 | 1256 | ACGUUGGGUACGAAUAUCUACGGC |
| osa-miR1431 | *OsCHX3* | 21 | 552 | 572 | UUUGCGAGUUGGCCCGCUUGC |
| osa-miR1437b-3p | *OsCHX14* | 24 | 558 | 581 | GUGCUGGCGAGCUCCGGUGCCGCA |
| osa-miR156c-3p | *OsKEA3* | 22 | 279 | 300 | GCUCACUUCUCUCUCUGUCAGC |
| osa-miR156g-3p | *OsKEA3* | 22 | 279 | 300 | GCUCACUUCUCUCUCUGUCAGC |
| osa-miR160d-3p | *OsKEA4* | 21 | 360 | 380 | GCGUGCGAGGAGCCAAGCAUG |
| osa-miR160f-3p | *OsNHX7* | 21 | 1836 | 1856 | GCAUUGAGGGAGUCAUGCAGG |
| osa-miR166h-5p | *OsCHX8* | 21 | 461 | 481 | GGAAUGUUGGCUGGCUCGAGG |
| osa-miR167a-5p | *OsNHX4* | 21 | 1372 | 1392 | UGAAGCUGCCAGCAUGAUCUA |
| osa-miR167b | *OsNHX4* | 21 | 1372 | 1392 | UGAAGCUGCCAGCAUGAUCUA |
| osa-miR167c-5p | *OsNHX4* | 21 | 1372 | 1392 | UGAAGCUGCCAGCAUGAUCUA |
| osa-miR167d-3p | *OsCHX12* | 22 | 177 | 198 | GAUCAUGCUGUGCAGUUUCAUC |
| osa-miR167d-5p | *OsNHX4* | 21 | 1372 | 1392 | UGAAGCUGCCAGCAUGAUCUG |
| osa-miR167e-3p | *OsNHX1* | 21 | 98 | 118 | AGAUCAUGUUGCAGCUUCACU |
| osa-miR167e-5p | *OsNHX4* | 21 | 1372 | 1392 | UGAAGCUGCCAGCAUGAUCUG |
| osa-miR167f | *OsNHX4* | 21 | 1372 | 1392 | UGAAGCUGCCAGCAUGAUCUG |
| osa-miR167g | *OsNHX4* | 21 | 1372 | 1392 | UGAAGCUGCCAGCAUGAUCUG |
| osa-miR167h-5p | *OsNHX4* | 21 | 1372 | 1392 | UGAAGCUGCCAGCAUGAUCUG |
| osa-miR167i-3p | *OsNHX1* | 21 | 98 | 118 | AGAUCAUGUUGCAGCUUCACU |
| osa-miR167i-5p | *OsNHX4* | 21 | 1372 | 1392 | UGAAGCUGCCAGCAUGAUCUG |
| osa-miR167j | *OsNHX4* | 21 | 1372 | 1392 | UGAAGCUGCCAGCAUGAUCUG |
| osa-miR169f.2 | *OsKEA3* | 21 | 1613 | 1633 | UGAGGACAAGAGCUGAUUCGG |
| osa-miR169n | *OsCHX18* | 21 | 1205 | 1225 | UAGCCAAGAAUGACUUGCCUA |
| osa-miR169o | *OsCHX18* | 21 | 1205 | 1225 | UAGCCAAGAAUGACUUGCCUA |
| osa-miR171f-5p | *OsKEA1* | 21 | 335 | 355 | UGUUGGCAUGGUUCAAUCAAA |
| osa-miR172c | *OsCHX8* | 21 | 2142 | 2162 | UGAAUCUUGAUGAUGCUGCAC |
| osa-miR1846a-5p | *OsCHX1* | 21 | 1198 | 1218 | AGUGAGGAGGCCGGGGCCGCU |
| osa-miR1846b-5p | *OsCHX1* | 21 | 1198 | 1218 | AGUGAGGAGGCCGGGGCCGCU |
| osa-miR1846c-5p | *OsCHX1* | 21 | 1198 | 1218 | AGUGAGGAGGCCGGGGCCGCU |
| osa-miR1848 | *OsCHX12* | 21 | 2245 | 2265 | CCUCGCCGGCGCGCGCGUGCA |
| osa-miR1848 | *OsCHX9* | 21 | 1741 | 1761 | CCUCGCCGGCGCGCGCGUGCA |
| osa-miR1848 | *OsCHX3* | 21 | 1741 | 1760 | CCUCGCCGGCGCGCGCGUGCA |
| osa-miR1855 | *OsKEA2* | 21 | 2391 | 2411 | AGCACUGGAGUAGCCAAGAGA |
| osa-miR1858a | *OsCHX4* | 21 | 1833 | 1853 | GAGAGGAGGACGGAGUGGGGC |
| osa-miR1858a | *OsNHX1* | 21 | 827 | 847 | GAGAGGAGGACGGAGUGGGGC |
| osa-miR1858a | *OsCHX17* | 21 | 288 | 308 | GAGAGGAGGACGGAGUGGGGC |
| osa-miR1858b | *OsCHX4* | 21 | 1833 | 1853 | GAGAGGAGGACGGAGUGGGGC |
| osa-miR1858b | *OsNHX1* | 21 | 827 | 847 | GAGAGGAGGACGGAGUGGGGC |
| osa-miR1858b | *OsCHX17* | 21 | 288 | 308 | GAGAGGAGGACGGAGUGGGGC |
| osa-miR1863a | *OsCHX5* | 24 | 945 | 968 | AGCUCUGAUACCAUGUUAGAUUAG |
| osa-miR1863b | *OsCHX5* | 24 | 945 | 968 | AGCUCUGAUACCAUGUUAACUGUU |
| osa-miR1872 | *OsCHX3* | 24 | 1015 | 1038 | GAACUGUAAGUCUGUGACGGGUAA |
| osa-miR2055 | *OsKEA4* | 21 | 1320 | 1340 | UUUCCUUGGGAAGGUGGUUUC |
| osa-miR2099-5p | *OsNHX7* | 22 | 1997 | 2018 | UGAAUAUGUUUGUACAAGCUUU |
| osa-miR2102-3p | *OsCHX18* | 22 | 1799 | 1820 | CAUGGUGCCGGUUCCGGUGGCG |
| osa-miR2104 | *OsCHX6* | 22 | 695 | 715 | GCGGCGAGGGGAUGCGAGCGUG |
| osa-miR2867-3p | *OsCHX13* | 20 | 108 | 127 | CCAGGACGUGUGGGAUGGCA |
| osa-miR2867-3p | *OsCHX15* | 20 | 108 | 127 | CCAGGACGUGUGGGAUGGCA |
| osa-miR2918 | *OsCHX16* | 21 | 1983 | 2003 | AUCCGUGUUGUCUGCGCUUUA |
| osa-miR2926 | *OsCHX11* | 20 | 2101 | 2120 | AGGUCGUCGACGUUGGUGCU |
| osa-miR2926 | *OsNHX6* | 20 | 100 | 119 | AGGUCGUCGACGUUGGUGCU |
| osa-miR2926 | *OsCHX1* | 20 | 2571 | 2590 | AGGUCGUCGACGUUGGUGCU |
| osa-miR2927 | *OsCHX12* | 23 | 1506 | 1528 | UGUCGUCGUCGAUGGAGCCCAUG |
| osa-miR395b | *OsNHX7* | 21 | 1939 | 1959 | GUGAAGUGUUUGGGGGAACUC |
| osa-miR395d | *OsNHX7* | 21 | 1939 | 1959 | GUGAAGUGUUUGGGGGAACUC |
| osa-miR395e | *OsNHX7* | 21 | 1939 | 1959 | GUGAAGUGUUUGGGGGAACUC |
| osa-miR395g | *OsNHX7* | 21 | 1939 | 1959 | GUGAAGUGUUUGGGGGAACUC |
| osa-miR395h | *OsNHX7* | 21 | 1939 | 1959 | GUGAAGUGUUUGGGGGAACUC |
| osa-miR395i | *OsNHX7* | 21 | 1939 | 1959 | GUGAAGUGUUUGGGGGAACUC |
| osa-miR395j | *OsNHX7* | 21 | 1939 | 1959 | GUGAAGUGUUUGGGGGAACUC |
| osa-miR395k | *OsNHX7* | 21 | 1939 | 1959 | GUGAAGUGUUUGGGGGAACUC |
| osa-miR395l | *OsNHX7* | 21 | 1939 | 1959 | GUGAAGUGUUUGGGGGAACUC |
| osa-miR395m | *OsNHX7* | 21 | 1939 | 1959 | GUGAAGUGUUUGGGGGAACUC |
| osa-miR395n | *OsNHX7* | 21 | 1939 | 1959 | GUGAAGUGUUUGGGGGAACUC |
| osa-miR395p | *OsNHX7* | 21 | 1939 | 1959 | GUGAAGUGUUUGGGGGAACUC |
| osa-miR395q | *OsNHX7* | 21 | 1939 | 1959 | GUGAAGUGUUUGGGGGAACUC |
| osa-miR395r | *OsNHX7* | 21 | 1939 | 1959 | GUGAAGUGUUUGGGGGAACUC |
| osa-miR395s | *OsNHX7* | 21 | 1939 | 1959 | GUGAAGUGUUUGGGGGAACUC |
| osa-miR395w | *OsNHX7* | 22 | 1938 | 1959 | GUGAAGUGUUUGGGGGAUUCUC |
| osa-miR395y | *OsNHX7* | 21 | 1939 | 1959 | GUGAAGUGUUUGGGGGAACUC |
| osa-miR414 | *OsCHX11* | 21 | 977 | 997 | UCAUCCUCAUCAUCAUCGUCC |
| osa-miR444b.2 | *OsKEA2* | 21 | 892 | 912 | UGCAGUUGUUGUCUCAAGCUU |
| osa-miR444c.2 | *OsKEA2* | 21 | 892 | 912 | UGCAGUUGUUGUCUCAAGCUU |
| osa-miR5074 | *OsNHX2* | 21 | 853 | 873 | GAAGGCCACCGUCGGGAUCGC |
| osa-miR5075 | *OsCHX4* | 21 | 2124 | 2144 | UUCUCCGUCGCCGCCGUCCGC |
| osa-miR5075 | *OsCHX12* | 21 | 1854 | 1874 | UUCUCCGUCGCCGCCGUCCGC |
| osa-miR5075 | *OsCHX13* | 21 | 2221 | 2241 | UUCUCCGUCGCCGCCGUCCGC |
| osa-miR5075 | *OsCHX15* | 21 | 2182 | 2202 | UUCUCCGUCGCCGCCGUCCGC |
| osa-miR5075 | *OsCHX5* | 21 | 2214 | 2234 | UUCUCCGUCGCCGCCGUCCGC |
| osa-miR5075 | *OsCHX3* | 21 | 2067 | 2087 | UUCUCCGUCGCCGCCGUCCGC |
| osa-miR5075 | *OsCHX11* | 21 | 1519 | 1539 | UUCUCCGUCGCCGCCGUCCGC |
| osa-miR5075 | *OsKEA3* | 21 | 96 | 116 | UUCUCCGUCGCCGCCGUCCGC |
| osa-miR5075 | *OsKEA3* | 21 | 117 | 137 | UUCUCCGUCGCCGCCGUCCGC |
| osa-miR5075 | *OsCHX18* | 21 | 1423 | 1443 | UUCUCCGUCGCCGCCGUCCGC |
| osa-miR5077 | *OsCHX17* | 19 | 1904 | 1922 | GUUCGCGUCGGGUUCACCA |
| osa-miR5082 | *OsCHX17* | 24 | 1666 | 1689 | UGCGAUGAUGGCCGCGCGGGUUCA |
| osa-miR5082 | *OsCHX10* | 24 | 1174 | 1197 | UGCGAUGAUGGCCGCGCGGGUUCA |
| osa-miR5082 | *OsCHX3* | 24 | 970 | 993 | UGCGAUGAUGGCCGCGCGGGUUCA |
| osa-miR5149 | *OsCHX4* | 22 | 975 | 996 | GAGGAGCUGUGACGAUUUGGGA |
| osa-miR528-5p | *OsNHX6* | 21 | 471 | 491 | UGGAAGGGGCAUGCAGAGGAG |
| osa-miR529b | *OsCHX14* | 21 | 965 | 985 | AGAAGAGAGAGAGUACAGCUU |
| osa-miR529b | *OsCHX16* | 21 | 1016 | 1036 | AGAAGAGAGAGAGUACAGCUU |
| osa-miR531a | *OsCHX9* | 24 | 2058 | 2081 | CUCGCCGGGGCUGCGUGCCGCCAU |
| osa-miR531b | *OsCHX9* | 20 | 2062 | 2081 | CUCGCCGGGGCUGCGUGCCG |
| osa-miR531c | *OsCHX9* | 24 | 2058 | 2081 | CUCGCCGGGGCUGCGUGCCGCCAU |
| osa-miR5492 | *OsNHX5* | 21 | 609 | 629 | AGAAGGAGAAUAGAUAUGGUU |
| osa-miR5500 | *OsKEA2* | 22 | 1041 | 1062 | AUCACUGAUGAAAUCUUGCGGC |
| osa-miR5505 | *OsKEA3* | 22 | 604 | 625 | GAGGAUUCGGUAUUGAUCGCUA |
| osa-miR5525 | *OsCHX10* | 22 | 553 | 574 | UGAACCUUGGGAGCGAUCUGAA |
| osa-miR5529 | *OsKEA2* | 21 | 576 | 596 | GUUUCAUCCAUGGACACCGCA |
| osa-miR5544 | *OsCHX9* | 22 | 952 | 973 | AGAACACGGAGUAGAAGUUGGU |
| osa-miR5809 | *OsCHX1* | 20 | 1305 | 1324 | UCGUCGCCGGCGACCACAGC |
| osa-miR5809 | *OsCHX13* | 20 | 2241 | 2260 | UCGUCGCCGGCGACCACAGC |
| osa-miR5809 | *OsCHX15* | 20 | 2202 | 2221 | UCGUCGCCGGCGACCACAGC |
| osa-miR5809 | *OsCHX9* | 20 | 315 | 334 | UCGUCGCCGGCGACCACAGC |
| osa-miR5819 | *OsCHX13* | 21 | 726 | 746 | AGGACGAGGGGAACGGCGGCG |
| osa-miR5819 | *OsNHX7* | 21 | 42 | 62 | AGGACGAGGGGAACGGCGGCG |
| osa-miR5819 | *OsCHX6* | 21 | 396 | 416 | AGGACGAGGGGAACGGCGGCG |
| osa-miR5819 | *OsKEA3* | 21 | 37 | 56 | AGGACGAGGGGAACGGCGGCG |
| osa-miR5821 | *OsKEA2* | 21 | 2251 | 2271 | UGGACGGAGCGAUGGUGGGCG |
| osa-miR812o-5p | *OsKEA2* | 21 | 1091 | 1111 | CGUGUUCAACGUUUGACUGUC |
| osa-miR812v | *OsNHX1* | 24 | 1020 | 1043 | AUGGCUGCACUUAAAAUGGGACGG |
| osa-miR815a | *OsNHX1* | 21 | 1312 | 1335 | AAGGGGAUUGAGGAGA---UUGGG |
| osa-miR815b | *OsNHX1* | 21 | 1312 | 1335 | AAGGGGAUUGAGGAGA---UUGGG |
| osa-miR815c | *OsNHX1* | 21 | 1312 | 1335 | AAGGGGAUUGAGGAGA---UUGGG |
| osa-miR1423-3p | *OsCHX4* | 21 | 568 | 587 | AGCGCCCAAGCGGUAGUUGUC |
| osa-miR1425-3p | *OsCHX8* | 21 | 2041 | 2061 | CAGCAAGAACUGGAUCUUAAU |
| osa-miR1425-5p | *OsKEA1* | 21 | 176 | 196 | UAGGAUUCAAUCCUUGCUGCU |
| osa-miR1427 | *OsCHX8* | 21 | 1751 | 1771 | UGCGGAACCGUGCGGUGGCGC |
| osa-miR1429-5p | *OsKEA2* | 21 | 2630 | 2650 | GUAAUAUACUAAUCCGUGCAU |
| osa-miR1430 | *OsKEA4* | 21 | 2148 | 2169 | UGGUGAGCCUUCCU-GGCUAAG |
| osa-miR1431 | *OsCHX13* | 21 | 56 | 76 | UUUGCGAGUUGGCCCGCUUGC |
| osa-miR1431 | *OsCHX15* | 21 | 56 | 76 | UUUGCGAGUUGGCCCGCUUGC |
| osa-miR1437b-3p | *OsCHX5* | 24 | 1965 | 1988 | GUGCUGGCGAGCUCCGGUGCCGCA |
| osa-miR1437b-3p | *OsCHX1* | 24 | 591 | 614 | GUGCUGGCGAGCUCCGGUGCCGCA |
| osa-miR1437b-3p | *OsCHX17* | 24 | 510 | 533 | GUGCUGGCGAGCUCCGGUGCCGCA |
| osa-miR156c-3p | *OsCHX3* | 22 | 2160 | 2181 | GCUCACUUCUCUCUCUGUCAGC |
| osa-miR156c-3p | *OsCHX12* | 22 | 2202 | 2223 | GCUCACUUCUCUCUCUGUCAGC |
| osa-miR156f-3p | *OsKEA3* | 22 | 279 | 300 | GCUCACUUCUCUUUCUGUCAGC |
| osa-miR156g-3p | *OsCHX3* | 22 | 2160 | 2181 | GCUCACUUCUCUCUCUGUCAGC |
| osa-miR156g-3p | *OsCHX12* | 22 | 2202 | 2223 | GCUCACUUCUCUCUCUGUCAGC |
| osa-miR156h-3p | *OsKEA3* | 22 | 279 | 300 | GCUCACUUCUCUUUCUGUCAGC |
| osa-miR156j-3p | *OsCHX18* | 22 | 2105 | 2126 | GCUCGCUCCUCUUUCUGUCAGC |
| osa-miR156j-3p | *OsCHX11* | 22 | 1280 | 1301 | GCUCGCUCCUCUUUCUGUCAGC |
| osa-miR156l-3p | *OsKEA3* | 22 | 279 | 300 | GCUCACUUCUCUUUCUGUCAGC |
| osa-miR160a-3p | *OsNHX1* | 21 | 1544 | 1564 | GCGUGCAAGGAGCCAAGCAUG |
| osa-miR160a-5p | *OsCHX16* | 21 | 2162 | 2182 | UGCCUGGCUCCCUGUAUGCCA |
| osa-miR160a-5p | *OsCHX14* | 21 | 2111 | 2131 | UGCCUGGCUCCCUGUAUGCCA |
| osa-miR160b-3p | *OsNHX1* | 21 | 1544 | 1564 | GCGUGCAAGGAGCCAAGCAUG |
| osa-miR160b-5p | *OsCHX16* | 21 | 2162 | 2182 | UGCCUGGCUCCCUGUAUGCCA |
| osa-miR160b-5p | *OsCHX14* | 21 | 2111 | 2131 | UGCCUGGCUCCCUGUAUGCCA |
| osa-miR160c-3p | *OsCHX12* | 21 | 1326 | 1346 | GCGUGCACGGAGCCAAGCAUA |
| osa-miR160c-3p | *OsCHX12* | 21 | 2403 | 2423 | GCGUGCACGGAGCCAAGCAUA |
| osa-miR160c-5p | *OsCHX16* | 21 | 2162 | 2182 | UGCCUGGCUCCCUGUAUGCCA |
| osa-miR160c-5p | *OsCHX14* | 21 | 2111 | 2131 | UGCCUGGCUCCCUGUAUGCCA |
| osa-miR160d-5p | *OsCHX16* | 21 | 2162 | 2182 | UGCCUGGCUCCCUGUAUGCCA |
| osa-miR160d-5p | *OsCHX14* | 21 | 2111 | 2131 | UGCCUGGCUCCCUGUAUGCCA |
| osa-miR160e-5p | *OsCHX16* | 21 | 2162 | 2182 | UGCCUGGCUCCCUGUAUGCCG |
| osa-miR160e-5p | *OsCHX14* | 21 | 2111 | 2131 | UGCCUGGCUCCCUGUAUGCCG |
| osa-miR164a | *OsNHX7* | 21 | 2423 | 2443 | UGGAGAAGCAGGGCACGUGCA |
| osa-miR164b | *OsNHX7* | 21 | 2423 | 2443 | UGGAGAAGCAGGGCACGUGCA |
| osa-miR164d | *OsNHX7* | 21 | 2423 | 2443 | UGGAGAAGCAGGGCACGUGCU |
| osa-miR164e | *OsNHX7* | 21 | 2423 | 2443 | UGGAGAAGCAGGGCACGUGAG |
| osa-miR164f | *OsNHX7* | 21 | 2423 | 2443 | UGGAGAAGCAGGGCACGUGCA |
| osa-miR166a-5p | *OsKEA2* | 21 | 1017 | 1037 | GGAAUGUUGUCUGGUUCAAGG |
| osa-miR166e-5p | *OsKEA2* | 21 | 1017 | 1037 | GGAAUGUUGUCUGGUUCAAGG |
| osa-miR167c-3p | *OsCHX5* | 23 | 2415 | 2437 | GGUCAUGCUGCGGCAGCCUCACU |
| osa-miR169e | *OsCHX18* | 21 | 1205 | 1225 | UAGCCAAGGAUGACUUGCCGG |
| osa-miR169f.1 | *OsCHX18* | 21 | 1205 | 1225 | UAGCCAAGGAUGACUUGCCUA |
| osa-miR169f.2 | *OsKEA4* | 21 | 701 | 721 | UGAGGACAAGAGCUGAUUCGG |
| osa-miR169f.2 | *OsKEA2* | 21 | 1997 | 2016 | UGAGGACAAGAGCUGAUUCGG |
| osa-miR169g | *OsCHX18* | 21 | 1205 | 1225 | UAGCCAAGGAUGACUUGCCUA |
| osa-miR169h | *OsCHX18* | 21 | 1205 | 1225 | UAGCCAAGGAUGACUUGCCUG |
| osa-miR169i-5p.1 | *OsCHX18* | 21 | 1205 | 1225 | UAGCCAAGGAUGACUUGCCUG |
| osa-miR169i-5p.2 | *OsCHX13* | 22 | 487 | 508 | UGGUGAUAAGGGUGUAGCUCUG |
| osa-miR169i-5p.2 | *OsCHX15* | 22 | 487 | 508 | UGGUGAUAAGGGUGUAGCUCUG |
| osa-miR169j | *OsCHX18* | 21 | 1205 | 1225 | UAGCCAAGGAUGACUUGCCUG |
| osa-miR169k | *OsCHX18* | 21 | 1205 | 1225 | UAGCCAAGGAUGACUUGCCUG |
| osa-miR169l | *OsCHX18* | 21 | 1205 | 1225 | UAGCCAAGGAUGACUUGCCUG |
| osa-miR169m | *OsCHX18* | 21 | 1205 | 1225 | UAGCCAAGGAUGACUUGCCUG |
| osa-miR171c-5p | *OsKEA4* | 21 | 1597 | 1617 | GGAUAUUGGUGCGGUUCAAUC |
| osa-miR172b | *OsNHX4* | 21 | 473 | 493 | GGAAUCUUGAUGAUGCUGCAU |
| osa-miR172d-5p | *OsNHX2* | 20 | 852 | 871 | GCAGCACCAUCAAGAUUCAC |
| osa-miR1846a-5p | *OsCHX6* | 21 | 814 | 834 | AGUGAGGAGGCCGGGGCCGCU |
| osa-miR1846a-5p | *OsCHX15* | 21 | 724 | 744 | AGUGAGGAGGCCGGGGCCGCU |
| osa-miR1846a-5p | *OsCHX16* | 21 | 271 | 291 | AGUGAGGAGGCCGGGGCCGCU |
| osa-miR1846a-5p | *OsCHX17* | 21 | 811 | 831 | AGUGAGGAGGCCGGGGCCGCU |
| osa-miR1846b-5p | *OsCHX6* | 21 | 814 | 834 | AGUGAGGAGGCCGGGGCCGCU |
| osa-miR1846b-5p | *OsCHX15* | 21 | 724 | 744 | AGUGAGGAGGCCGGGGCCGCU |
| osa-miR1846b-5p | *OsCHX16* | 21 | 271 | 291 | AGUGAGGAGGCCGGGGCCGCU |
| osa-miR1846b-5p | *OsCHX17* | 21 | 811 | 831 | AGUGAGGAGGCCGGGGCCGCU |
| osa-miR1846c-5p | *OsCHX6* | 21 | 814 | 834 | AGUGAGGAGGCCGGGGCCGCU |
| osa-miR1846c-5p | *OsCHX15* | 21 | 724 | 744 | AGUGAGGAGGCCGGGGCCGCU |
| osa-miR1846c-5p | *OsCHX16* | 21 | 271 | 291 | AGUGAGGAGGCCGGGGCCGCU |
| osa-miR1846c-5p | *OsCHX17* | 21 | 811 | 831 | AGUGAGGAGGCCGGGGCCGCU |
| osa-miR1847.1 | *OsCHX5* | 21 | 1329 | 1349 | UGCAGUUUGCAGUUGUGGCAC |
| osa-miR1847.2 | *OsNHX7* | 24 | 185 | 208 | UGGCCCACAUGUUAGUGCCACAAC |
| osa-miR1848 | *OsCHX12* | 21 | 1347 | 1368 | CCUCGCCGGCGCG-CGCGUGCA |
| osa-miR1848 | *OsCHX12* | 21 | 2269 | 2289 | CCUCGCCGGCGCGCGCGUGCA |
| osa-miR1848 | *OsCHX10* | 21 | 1585 | 1605 | CCUCGCCGGCGCGCGCGUGCA |
| osa-miR1848 | *OsCHX3* | 21 | 1603 | 1623 | CCUCGCCGGCGCGCGCGUGCA |
| osa-miR1848 | *OsCHX8* | 21 | 1742 | 1762 | CCUCGCCGGCGCGCGCGUGCA |
| osa-miR1848 | *OsCHX11* | 21 | 928 | 948 | CCUCGCCGGCGCGCGCGUGCA |
| osa-miR1848 | *OsCHX4* | 21 | 1488 | 1508 | CCUCGCCGGCGCGCGCGUGCA |
| osa-miR1853-3p | *OsCHX10* | 22 | 1843 | 1865 | UAAUUGGGGAUGUUCG-GUUGCU |
| osa-miR1858a | *OsCHX15* | 21 | 321 | 341 | GAGAGGAGGACGGAGUGGGGC |
| osa-miR1858a | *OsCHX13* | 21 | 321 | 341 | GAGAGGAGGACGGAGUGGGGC |
| osa-miR1858a | *OsCHX16* | 21 | 199 | 219 | GAGAGGAGGACGGAGUGGGGC |
| osa-miR1858a | *OsCHX18* | 21 | 309 | 329 | GAGAGGAGGACGGAGUGGGGC |
| osa-miR1858b | *OsCHX13* | 21 | 321 | 341 | GAGAGGAGGACGGAGUGGGGC |
| osa-miR1858b | *OsCHX15* | 21 | 321 | 341 | GAGAGGAGGACGGAGUGGGGC |
| osa-miR1858b | *OsCHX16* | 21 | 199 | 219 | GAGAGGAGGACGGAGUGGGGC |
| osa-miR1858b | *OsCHX18* | 21 | 309 | 329 | GAGAGGAGGACGGAGUGGGGC |
| osa-miR1863a | *OsCHX14* | 24 | 1344 | 1367 | AGCUCUGAUACCAUGUUAGAUUAG |
| osa-miR1865-5p | *OsNHX1* | 24 | 1174 | 1197 | UGCUAGUGAUGGUGAUUCUUCGAC |
| osa-miR1866-3p | *OsNHX3* | 22 | 307 | 328 | UGAAAUUCCUGUAAAAUUCUUG |
| osa-miR1870-3p | *OsKEA3* | 24 | 1791 | 1814 | UUUAGGGCUAAUUCAGCAUGAACA |
| osa-miR1871 | *OsCHX9* | 24 | 302 | 325 | AUGGCUCUGAUAUCAUGUUGGUUU |
| osa-miR1871 | *OsCHX5* | 24 | 947 | 970 | AUGGCUCUGAUAUCAUGUUGGUUU |
| osa-miR1874-3p | *OsCHX1* | 24 | 1679 | 1702 | UAUGGAUGGAGGUGUAACCCGAUG |
| osa-miR2055 | *OsNHX7* | 21 | 260 | 280 | UUUCCUUGGGAAGGUGGUUUC |
| osa-miR2055 | *OsNHX4* | 21 | 67 | 87 | UUUCCUUGGGAAGGUGGUUUC |
| osa-miR2095-5p | *OsKEA2* | 22 | 973 | 994 | CUGAUAAUUUUACGAUGAAUAG |
| osa-miR2097-3p | *OsKEA3* | 22 | 170 | 191 | UUCUCUUCUUCGUGUCGCAUUU |
| osa-miR2098-3p | *OsCHX8* | 20 | 758 | 777 | CGGUUUGUCAAGCGGAGUGC |
| osa-miR2099-5p | *OsNHX5* | 22 | 439 | 460 | UGAAUAUGUUUGUACAAGCUUU |
| osa-miR2102-3p | *OsCHX8* | 22 | 1509 | 1530 | CAUGGUGCCGGUUCCGGUGGCG |
| osa-miR2102-3p | *OsCHX9* | 22 | 906 | 927 | CAUGGUGCCGGUUCCGGUGGCG |
| osa-miR2102-3p | *OsCHX18* | 22 | 552 | 573 | CAUGGUGCCGGUUCCGGUGGCG |
| osa-miR2102-3p | *OsCHX1* | 22 | 876 | 897 | CAUGGUGCCGGUUCCGGUGGCG |
| osa-miR2102-3p | *OsCHX3* | 22 | 66 | 87 | CAUGGUGCCGGUUCCGGUGGCG |
| osa-miR2102-5p | *OsCHX3* | 20 | 2249 | 2268 | GGGCAAGCCGCCGCCGCCAC |
| osa-miR2102-5p | *OsCHX13* | 20 | 31 | 50 | GGGCAAGCCGCCGCCGCCAC |
| osa-miR2102-5p | *OsCHX15* | 20 | 31 | 50 | GGGCAAGCCGCCGCCGCCAC |
| osa-miR2103 | *OsCHX5* | 22 | 901 | 922 | UUUCCCUCUCCGUGCGCGCUCG |
| osa-miR2104 | *OsCHX10* | 22 | 441 | 463 | GCGGCGAGGGGAUG-CGAGCGUG |
| osa-miR2105 | *OsNHX7* | 20 | 2882 | 2901 | UUGUGAUGUGAAUGAUUCAU |
| osa-miR2106 | *OsKEA2* | 21 | 343 | 362 | CCGAGGUUUUCUGGAUACAUU |
| osa-miR2118c | *OsCHX18* | 22 | 1534 | 1555 | UUCCCGAUGCCUCCUAUUCCUA |
| osa-miR2118q | *OsCHX18* | 22 | 1534 | 1555 | UUCCCGAUGCCUCCUAUUCCUA |
| osa-miR2866 | *OsCHX16* | 20 | 2181 | 2200 | UCUAGUUUGUGUUCAGCAUC |
| osa-miR2866 | *OsCHX14* | 20 | 2130 | 2149 | UCUAGUUUGUGUUCAGCAUC |
| osa-miR2870 | *OsKEA3* | 21 | 1211 | 1231 | UAAUCAGUUUGGGGAGACAAA |
| osa-miR2918 | *OsCHX17* | 21 | 1902 | 1922 | AUCCGUGUUGUCUGCGCUUUA |
| osa-miR2919 | *OsCHX1* | 19 | 161 | 179 | AAGGGGGGGGGGGGAAAGA |
| osa-miR2919 | *OsCHX10* | 19 | 140 | 158 | AAGGGGGGGGGGGGAAAGA |
| osa-miR2919 | *OsCHX13* | 19 | 522 | 540 | AAGGGGGGGGGGGGAAAGA |
| osa-miR2919 | *OsCHX15* | 19 | 522 | 540 | AAGGGGGGGGGGGGAAAGA |
| osa-miR2919 | *OsCHX4* | 19 | 266 | 284 | AAGGGGGGGGGGGGAAAGA |
| osa-miR2919 | *OsCHX14* | 19 | 92 | 110 | AAGGGGGGGGGGGGAAAGA |
| osa-miR2919 | *OsCHX16* | 19 | 143 | 161 | AAGGGGGGGGGGGGAAAGA |
| osa-miR2924 | *OsCHX11* | 21 | 1197 | 1217 | CUCGCUUGCUCCGGCCGCCAC |
| osa-miR2924 | *OsKEA3* | 21 | 97 | 117 | CUCGCUUGCUCCGGCCGCCAC |
| osa-miR2925 | *OsCHX3* | 19 | 1030 | 1048 | UGGCGGCCGCGGGCUUCGU |
| osa-miR2925 | *OsCHX6* | 19 | 580 | 598 | UGGCGGCCGCGGGCUUCGU |
| osa-miR2925 | *OsCHX4* | 19 | 547 | 565 | UGGCGGCCGCGGGCUUCGU |
| osa-miR2926 | *OsCHX11* | 20 | 778 | 797 | AGGUCGUCGACGUUGGUGCU |
| osa-miR2926 | *OsCHX5* | 20 | 2464 | 2483 | AGGUCGUCGACGUUGGUGCU |
| osa-miR2926 | *OsCHX8* | 20 | 582 | 601 | AGGUCGUCGACGUUGGUGCU |
| osa-miR2927 | *OsCHX12* | 23 | 2067 | 2089 | UGUCGUCGUCGAUGGAGCCCAUG |
| osa-miR2927 | *OsCHX5* | 23 | 1695 | 1717 | UGUCGUCGUCGAUGGAGCCCAUG |
| osa-miR2927 | *OsCHX5* | 23 | 2299 | 2322 | UGUCGUCGUCGAUGG-AGCCCAUG |
| osa-miR2927 | *OsKEA2* | 23 | 2360 | 2382 | UGUCGUCGUCGAUGGAGCCCAUG |
| osa-miR2927 | *OsCHX1* | 23 | 663 | 685 | UGUCGUCGUCGAUGGAGCCCAUG |
| osa-miR2927 | *OsCHX10* | 23 | 105 | 127 | UGUCGUCGUCGAUGGAGCCCAUG |
| osa-miR2927 | *OsCHX13* | 23 | 2240 | 2262 | UGUCGUCGUCGAUGGAGCCCAUG |
| osa-miR2927 | *OsCHX15* | 23 | 2201 | 2223 | UGUCGUCGUCGAUGGAGCCCAUG |
| osa-miR393b-3p | *OsKEA2* | 21 | 200 | 220 | UCAGUGCAAUCCCUUUGGAAU |
| osa-miR394 | *OsCHX17* | 20 | 2139 | 2158 | UUGGCAUUCUGUCCACCUCC |
| osa-miR394 | *OsCHX12* | 20 | 819 | 838 | UUGGCAUUCUGUCCACCUCC |
| osa-miR395o | *OsNHX7* | 21 | 1939 | 1959 | AUGAAGUGUUUGGAGGAACUC |
| osa-miR395t | *OsNHX1* | 21 | 729 | 749 | GUGAAGUGUUUGGGGAAACUC |
| osa-miR395v | *OsNHX7* | 21 | 1939 | 1959 | GUGAAGUAUUUGGCGGAACUC |
| osa-miR395w | *OsKEA4* | 22 | 2176 | 2197 | GUGAAGUGUUUGGGGGAUUCUC |
| osa-miR396f-5p | *OsCHX8* | 22 | 763 | 784 | UCUCCACAGGCUUUCUUGAACU |
| osa-miR3979-3p | *OsKEA2* | 20 | 1338 | 1357 | CUUCGGGGGAGGAGAGAAGC |
| osa-miR3979-3p | *OsCHX9* | 20 | 2397 | 2416 | CUUCGGGGGAGGAGAGAAGC |
| osa-miR3979-3p | *OsCHX2* | 20 | 666 | 685 | CUUCGGGGGAGGAGAGAAGC |
| osa-miR3979-3p | *OsNHX4* | 20 | 758 | 777 | CUUCGGGGGAGGAGAGAAGC |
| osa-miR397a | *OsCHX11* | 21 | 1208 | 1228 | UCAUUGAGUGCAGCGUUGAUG |
| osa-miR3980a-3p | *OsCHX13* | 21 | 172 | 192 | CUGGCCGAGGCCGUCGAUUCU |
| osa-miR3980a-3p | *OsCHX15* | 21 | 172 | 192 | CUGGCCGAGGCCGUCGAUUCU |
| osa-miR3980a-3p | *OsCHX7* | 21 | 271 | 291 | CUGGCCGAGGCCGUCGAUUCU |
| osa-miR3980a-5p | *OsCHX9* | 21 | 310 | 330 | AAUCGACGGCCUCAGUCAGGG |
| osa-miR3980b-3p | *OsCHX13* | 21 | 172 | 192 | CUGGCCGAGGCCGUCGAUUCU |
| osa-miR3980b-3p | *OsCHX15* | 21 | 172 | 192 | CUGGCCGAGGCCGUCGAUUCU |
| osa-miR3980b-3p | *OsCHX7* | 21 | 271 | 291 | CUGGCCGAGGCCGUCGAUUCU |
| osa-miR3980b-5p | *OsCHX9* | 21 | 310 | 330 | AAUCGACGGCCUCAGUCAGGG |
| osa-miR399i | *OsCHX8* | 21 | 1955 | 1975 | UGCCAAAGGAGAGCUGCCCUG |
| osa-miR399k | *OsNHX7* | 21 | 2639 | 2659 | UGCCAAAGGAAAUUUGCCCCG |
| osa-miR408-3p | *OsCHX3* | 21 | 2065 | 2085 | CUGCACUGCCUCUUCCCUGGC |
| osa-miR416 | *OsCHX8* | 21 | 320 | 340 | UGUUCGUCCGUACACUGUUCA |
| osa-miR419 | *OsCHX6* | 21 | 867 | 886 | UGAUGAAUGCUGACGAUGUUG |
| osa-miR440 | *OsCHX17* | 24 | 2243 | 2266 | AGUGUCUCCUGAUGAUCGGGACAA |
| osa-miR440 | *OsCHX18* | 24 | 1911 | 1934 | AGUGUCUCCUGAUGAUCGGGACAA |
| osa-miR440 | *OsCHX13* | 24 | 1938 | 1961 | AGUGUCUCCUGAUGAUCGGGACAA |
| osa-miR440 | *OsCHX15* | 24 | 1899 | 1922 | AGUGUCUCCUGAUGAUCGGGACAA |
| osa-miR444f | *OsKEA3* | 21 | 1504 | 1524 | UGCAGUUGUUGCCUCAAGCUU |
| osa-miR5071 | *OsNHX7* | 21 | 555 | 575 | UCAAGCAUCAUAUCGUGGACA |
| osa-miR5074 | *OsCHX18* | 21 | 637 | 657 | GAAGGCCACCGUCGGGAUCGC |
| osa-miR5074 | *OsKEA2* | 21 | 2450 | 2470 | GAAGGCCACCGUCGGGAUCGC |
| osa-miR5074 | *OsCHX13* | 21 | 1279 | 1299 | GAAGGCCACCGUCGGGAUCGC |
| osa-miR5075 | *OsCHX9* | 21 | 318 | 338 | UUCUCCGUCGCCGCCGUCCGC |
| osa-miR5075 | *OsCHX13* | 21 | 1641 | 1661 | UUCUCCGUCGCCGCCGUCCGC |
| osa-miR5075 | *OsCHX15* | 21 | 1602 | 1622 | UUCUCCGUCGCCGCCGUCCGC |
| osa-miR5075 | *OsCHX14* | 21 | 2229 | 2249 | UUCUCCGUCGCCGCCGUCCGC |
| osa-miR5075 | *OsCHX14* | 21 | 2406 | 2426 | UUCUCCGUCGCCGCCGUCCGC |
| osa-miR5075 | *OsCHX5* | 21 | 28 | 47 | UUCUCCGUCGCCGCCGUCCGC |
| osa-miR5075 | *OsCHX16* | 21 | 2457 | 2477 | UUCUCCGUCGCCGCCGUCCGC |
| osa-miR5075 | *OsKEA3* | 21 | 201 | 221 | UUCUCCGUCGCCGCCGUCCGC |
| osa-miR5075 | *OsCHX18* | 21 | 2203 | 2223 | UUCUCCGUCGCCGCCGUCCGC |
| osa-miR5075 | *OsCHX10* | 21 | 2092 | 2111 | UUCUCCGUCGCCGCCGUCCGC |
| osa-miR5075 | *OsCHX1* | 21 | 2343 | 2363 | UUCUCCGUCGCCGCCGUCCGC |
| osa-miR5075 | *OsCHX1* | 21 | 1335 | 1355 | UUCUCCGUCGCCGCCGUCCGC |
| osa-miR5075 | *OsCHX1* | 21 | 288 | 308 | UUCUCCGUCGCCGCCGUCCGC |
| osa-miR5075 | *OsKEA4* | 21 | 194 | 214 | UUCUCCGUCGCCGCCGUCCGC |
| osa-miR5075 | *OsNHX6* | 21 | 57 | 77 | UUCUCCGUCGCCGCCGUCCGC |
| osa-miR5077 | *OsCHX14* | 19 | 1295 | 1313 | GUUCGCGUCGGGUUCACCA |
| osa-miR5077 | *OsCHX16* | 19 | 1346 | 1364 | GUUCGCGUCGGGUUCACCA |
| osa-miR5082 | *OsCHX6* | 24 | 1024 | 1047 | UGCGAUGAUGGCCGCGCGGGUUCA |
| osa-miR5082 | *OsCHX12* | 24 | 67 | 90 | UGCGAUGAUGGCCGCGCGGGUUCA |
| osa-miR5143a | *OsNHX4* | 24 | 1161 | 1184 | UGUGGUAUGUUGGCAAUGUAGGAA |
| osa-miR5143a | *OsCHX18* | 24 | 20 | 43 | UGUGGUAUGUUGGCAAUGUAGGAA |
| osa-miR5143b | *OsNHX4* | 24 | 1161 | 1184 | UGUGGUAUGUUGGCAAUGUAGGAA |
| osa-miR5143b | *OsCHX18* | 24 | 20 | 43 | UGUGGUAUGUUGGCAAUGUAGGAA |
| osa-miR5144-5p | *OsNHX7* | 21 | 2195 | 2215 | UUCUUGUGCUGCUGAAGAGAC |
| osa-miR5148a | *OsKEA3* | 24 | 779 | 802 | UGAGGGGUAGAAAUGUCAUAUCAU |
| osa-miR5148a | *OsNHX6* | 24 | 929 | 952 | UGAGGGGUAGAAAUGUCAUAUCAU |
| osa-miR5148b | *OsKEA3* | 24 | 779 | 802 | UGAGGGGUAGAAAUGUCAUAUCAU |
| osa-miR5148b | *OsNHX6* | 24 | 929 | 952 | UGAGGGGUAGAAAUGUCAUAUCAU |
| osa-miR5148c | *OsKEA3* | 24 | 779 | 802 | UGAGGGGUAGAAAUGUCAUAUCAU |
| osa-miR5148c | *OsNHX6* | 24 | 929 | 952 | UGAGGGGUAGAAAUGUCAUAUCAU |
| osa-miR5149 | *OsKEA4* | 22 | 603 | 624 | GAGGAGCUGUGACGAUUUGGGA |
| osa-miR5149 | *OsCHX8* | 22 | 1021 | 1041 | GAGGAGCUGUGACGAUUUGGGA |
| osa-miR5158 | *OsCHX17* | 24 | 2358 | 2381 | UGAGCCACUGGGAUGAGGAUGAAU |
| osa-miR5159 | *OsCHX1* | 24 | 141 | 164 | AACUAGAGUGGGUCAACGGGUACC |
| osa-miR5160 | *OsCHX16* | 22 | 1213 | 1234 | CGAGAUCGAUGGUAUAUUUCUG |
| osa-miR5160 | *OsCHX14* | 22 | 1162 | 1183 | CGAGAUCGAUGGUAUAUUUCUG |
| osa-miR528-5p | *OsCHX5* | 21 | 691 | 711 | UGGAAGGGGCAUGCAGAGGAG |
| osa-miR529a | *OsCHX13* | 20 | 2145 | 2164 | CUGUACCCUCUCUCUUCUUC |
| osa-miR529a | *OsCHX15* | 20 | 2106 | 2125 | CUGUACCCUCUCUCUUCUUC |
| osa-miR530-3p | *OsNHX6* | 21 | 86 | 106 | AGGUGCAGAGGCAGAUGCAAC |
| osa-miR530-3p | *OsNHX5* | 21 | 538 | 558 | AGGUGCAGAGGCAGAUGCAAC |
| osa-miR531a | *OsCHX9* | 24 | 2306 | 2328 | CUCGCCGGGGCUGCGUGCCGCCAU |
| osa-miR531a | *OsCHX3* | 24 | 1819 | 1842 | CUCGCCGGGGCUGCGUGCCGCCAU |
| osa-miR531a | *OsCHX5* | 24 | 535 | 558 | CUCGCCGGGGCUGCGUGCCGCCAU |
| osa-miR531a | *OsCHX4* | 24 | 487 | 510 | CUCGCCGGGGCUGCGUGCCGCCAU |
| osa-miR531a | *OsCHX2* | 24 | 327 | 350 | CUCGCCGGGGCUGCGUGCCGCCAU |
| osa-miR531a | *OsCHX2* | 24 | 575 | 597 | CUCGCCGGGGCUGCGUGCCGCCAU |
| osa-miR531b | *OsCHX9* | 20 | 2310 | 2328 | CUCGCCGGGGCUGCGUGCCG |
| osa-miR531b | *OsCHX5* | 20 | 539 | 558 | CUCGCCGGGGCUGCGUGCCG |
| osa-miR531b | *OsCHX4* | 20 | 491 | 510 | CUCGCCGGGGCUGCGUGCCG |
| osa-miR531b | *OsCHX3* | 20 | 1823 | 1842 | CUCGCCGGGGCUGCGUGCCG |
| osa-miR531b | *OsCHX2* | 20 | 331 | 350 | CUCGCCGGGGCUGCGUGCCG |
| osa-miR531b | *OsCHX2* | 20 | 579 | 597 | CUCGCCGGGGCUGCGUGCCG |
| osa-miR531c | *OsCHX9* | 24 | 2306 | 2328 | CUCGCCGGGGCUGCGUGCCGCCAU |
| osa-miR531c | *OsCHX3* | 24 | 1819 | 1842 | CUCGCCGGGGCUGCGUGCCGCCAU |
| osa-miR531c | *OsCHX5* | 24 | 535 | 558 | CUCGCCGGGGCUGCGUGCCGCCAU |
| osa-miR531c | *OsCHX4* | 24 | 487 | 510 | CUCGCCGGGGCUGCGUGCCGCCAU |
| osa-miR531c | *OsCHX2* | 24 | 327 | 350 | CUCGCCGGGGCUGCGUGCCGCCAU |
| osa-miR531c | *OsCHX2* | 24 | 575 | 597 | CUCGCCGGGGCUGCGUGCCGCCAU |
| osa-miR5339 | *OsNHX6* | 21 | 637 | 657 | CAGAUAGAGAAUCUUCUCAGA |
| osa-miR535-5p | *OsNHX7* | 21 | 710 | 730 | UGACAACGAGAGAGAGCACGC |
| osa-miR5487 | *OsCHX8* | 21 | 784 | 804 | AAAGAUGUGCAUGUAGUUCCG |
| osa-miR5500 | *OsNHX6* | 22 | 601 | 621 | AUCACUGAUGAAAUCUUGCGGC |
| osa-miR5506 | *OsCHX6* | 21 | 45 | 65 | UGGAUCGCUUCGUCUGAUGGU |
| osa-miR5510 | *OsKEA4* | 21 | 520 | 540 | AGGCUGAUCCACUCCAGAGGA |
| osa-miR5511 | *OsCHX11* | 21 | 2253 | 2273 | CAUAUCCCAGCUGUUUCGGCC |
| osa-miR5526 | *OsNHX4* | 21 | 1551 | 1571 | AAAGGUAGAGUCAGGUAUGAG |
| osa-miR5536 | *OsCHX3* | 22 | 1992 | 2013 | AAUGGUAGUGACAUUAUGGUAG |
| osa-miR5538 | *OsNHX7* | 22 | 1156 | 1177 | ACUGAACUCAAUCACUUGCUGC |
| osa-miR5540 | *OsKEA2* | 21 | 3247 | 3267 | UUGUGCGAGAUCGACGGUAUA |
| osa-miR5789 | *OsCHX4* | 21 | 2163 | 2183 | UGACUGAGCUUCGUUCGGUAU |
| osa-miR5791 | *OsKEA3* | 21 | 1108 | 1128 | UUGCAGGAGACUAGAGACCAG |
| osa-miR5792 | *OsCHX12* | 24 | 64 | 87 | GAUGACAGCGGUGGUUCGGACAUC |
| osa-miR5795 | *OsCHX17* | 22 | 320 | 341 | AUGUCGAGGUCGAGUUCCCGGC |
| osa-miR5797 | *OsNHX4* | 21 | 1500 | 1520 | UCGUGGGAUUUAUGCAGUUAA |
| osa-miR5804 | *OsCHX5* | 21 | 970 | 990 | UGCGAAGUAGAGAUGCCGACU |
| osa-miR5806 | *OsNHX4* | 21 | 1141 | 1161 | ACAGGCAAAGACAAUGACGGC |
| osa-miR5809 | *OsCHX1* | 20 | 1329 | 1348 | UCGUCGCCGGCGACCACAGC |
| osa-miR5809 | *OsCHX9* | 20 | 2541 | 2560 | UCGUCGCCGGCGACCACAGC |
| osa-miR5809 | *OsCHX11* | 20 | 2338 | 2357 | UCGUCGCCGGCGACCACAGC |
| osa-miR5809 | *OsCHX8* | 20 | 1215 | 1234 | UCGUCGCCGGCGACCACAGC |
| osa-miR5819 | *OsCHX13* | 21 | 408 | 428 | AGGACGAGGGGAACGGCGGCG |
| osa-miR5819 | *OsCHX1* | 21 | 768 | 788 | AGGACGAGGGGAACGGCGGCG |
| osa-miR5819 | *OsCHX5* | 21 | 426 | 446 | AGGACGAGGGGAACGGCGGCG |
| osa-miR5819 | *OsCHX15* | 21 | 726 | 746 | AGGACGAGGGGAACGGCGGCG |
| osa-miR5819 | *OsCHX15* | 21 | 408 | 428 | AGGACGAGGGGAACGGCGGCG |
| osa-miR5819 | *OsCHX10* | 21 | 213 | 233 | AGGACGAGGGGAACGGCGGCG |
| osa-miR5819 | *OsNHX2* | 21 | 1394 | 1414 | AGGACGAGGGGAACGGCGGCG |
| osa-miR5821 | *OsNHX1* | 21 | 1268 | 1288 | UGGACGGAGCGAUGGUGGGCG |
| osa-miR5822 | *OsCHX12* | 21 | 603 | 623 | UGUCUGCUCGAUGUCAGGUUG |
| osa-miR5827 | *OsKEA3* | 21 | 750 | 770 | UUUGUUGCAAUUUGGACUACC |
| osa-miR5829 | *OsNHX7* | 24 | 310 | 333 | AUCAGGACCAGUAGGCGAUGGUAA |
| osa-miR5831 | *OsNHX1* | 24 | 1034 | 1057 | UAGUCAAACUUAGAAUAGUUGGAC |
| osa-miR5832 | *OsCHX3* | 21 | 768 | 788 | UUGGCGGAGCGGUUGCUGUCA |
| osa-miR5837.2 | *OsCHX16* | 21 | 160 | 180 | GGUGAUGUGGAGCGUUCGGCA |
| osa-miR5837.2 | *OsCHX14* | 21 | 109 | 129 | GGUGAUGUGGAGCGUUCGGCA |
| osa-miR6249a | *OsCHX11* | 21 | 2143 | 2163 | CGUGAAGAGCUCGCCGGCGGC |
| osa-miR6249a | *OsCHX3* | 21 | 463 | 483 | CGUGAAGAGCUCGCCGGCGGC |
| osa-miR6249b | *OsCHX11* | 21 | 2143 | 2163 | CGUGAAGAGCUCGCCGGCGGC |
| osa-miR6249b | *OsCHX3* | 21 | 463 | 483 | CGUGAAGAGCUCGCCGGCGGC |
| osa-miR7695-5p | *OsCHX11* | 24 | 1634 | 1657 | UGCCUAUGUGGCACGCCACGUGAA |
| osa-miR812n-5p | *OsNHX6* | 24 | 760 | 782 | AAGUGCAGCCAUGAGUUUCCGUGC |
| osa-miR812q | *OsCHX9* | 24 | 1264 | 1286 | ACGUUGGGUACGAAUAUCUACGGC |
| osa-miR815a | *OsCHX12* | 21 | 1369 | 1389 | AAGGGGAUUGAGGAGAUUGGG |
| osa-miR815a | *OsKEA2* | 21 | 2708 | 2728 | AAGGGGAUUGAGGAGAUUGGG |
| osa-miR815a | *OsKEA3* | 21 | 1176 | 1196 | AAGGGGAUUGAGGAGAUUGGG |
| osa-miR815a | *OsCHX9* | 21 | 253 | 273 | AAGGGGAUUGAGGAGAUUGGG |
| osa-miR815b | *OsCHX12* | 21 | 1369 | 1389 | AAGGGGAUUGAGGAGAUUGGG |
| osa-miR815b | *OsKEA2* | 21 | 2708 | 2728 | AAGGGGAUUGAGGAGAUUGGG |
| osa-miR815b | *OsKEA3* | 21 | 1176 | 1196 | AAGGGGAUUGAGGAGAUUGGG |
| osa-miR815b | *OsCHX9* | 21 | 253 | 273 | AAGGGGAUUGAGGAGAUUGGG |
| osa-miR815c | *OsCHX12* | 21 | 1369 | 1389 | AAGGGGAUUGAGGAGAUUGGG |
| osa-miR815c | *OsKEA2* | 21 | 2708 | 2728 | AAGGGGAUUGAGGAGAUUGGG |
| osa-miR815c | *OsKEA3* | 21 | 1176 | 1196 | AAGGGGAUUGAGGAGAUUGGG |
| osa-miR815c | *OsCHX9* | 21 | 253 | 273 | AAGGGGAUUGAGGAGAUUGGG |
